# Pharmacological Inhibition of Oncogenic STAT3 and STAT5 Signaling in Hematopoietic Cancers

**DOI:** 10.3390/cancers12010240

**Published:** 2020-01-18

**Authors:** Marie Brachet-Botineau, Marion Polomski, Heidi A. Neubauer, Ludovic Juen, Damien Hédou, Marie-Claude Viaud-Massuard, Gildas Prié, Fabrice Gouilleux

**Affiliations:** 1 Leukemic Niche and Oxidative metabolism (LNOx), CNRS ERL 7001, University of Tours, 37000 Tours, France; marie.brachet@univ-tours.fr; 2 Innovation Moléculaire et Thérapeutique (IMT), EA 7501, University of Tours, 37000 Tours, France; marion.polomski@etu.univ-tours.fr (M.P.); ludovic.juen@mcsaf.fr (L.J.); damien.hedou@univ-tours.fr (D.H.); marie-claude.viaud-massuard@univ-tours.fr (M.-C.V.-M.); gildas.prie@univ-tours.fr (G.P.); 3 Institute of Animal Breeding and Genetics, University of Veterinary Medicine Vienna, A-1210 Vienna, Austria; heidi.neubauer@vetmeduni.ac.at

**Keywords:** STAT3, STAT5, hematopoietic cancers, therapeutic targeting, pharmacological inhibitors

## Abstract

Signal Transducer and Activator of Transcription (STAT) 3 and 5 are important effectors of cellular transformation, and aberrant STAT3 and STAT5 signaling have been demonstrated in hematopoietic cancers. STAT3 and STAT5 are common targets for different tyrosine kinase oncogenes (TKOs). In addition, STAT3 and STAT5 proteins were shown to contain activating mutations in some rare but aggressive leukemias/lymphomas. Both proteins also contribute to drug resistance in hematopoietic malignancies and are now well recognized as major targets in cancer treatment. The development of inhibitors targeting STAT3 and STAT5 has been the subject of intense investigations during the last decade. This review summarizes the current knowledge of oncogenic STAT3 and STAT5 functions in hematopoietic cancers as well as advances in preclinical and clinical development of pharmacological inhibitors.

## 1. Introduction

**Figure 1:**
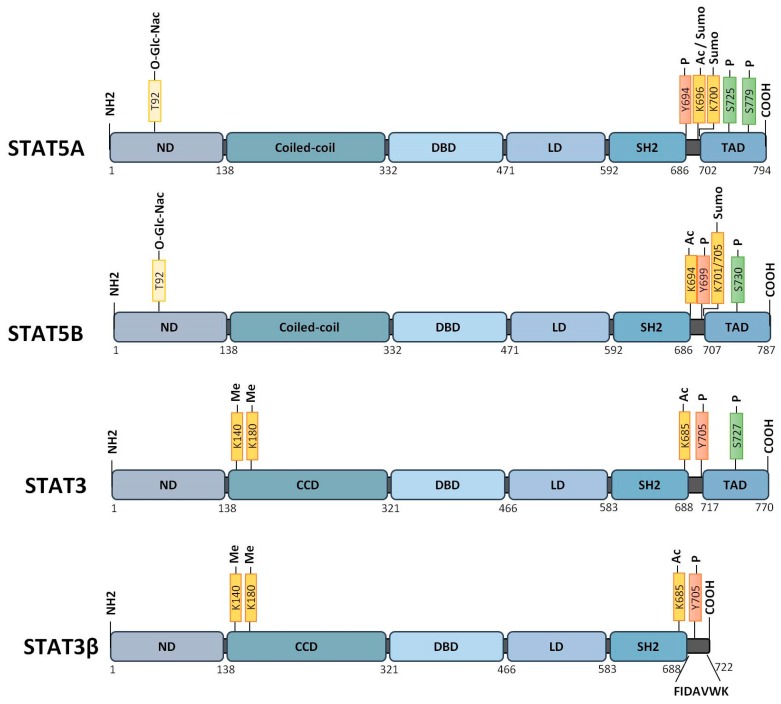
Structure of Signal Transducer and Activator of Transcription (STAT)5A, STAT5B, STAT3, and the spliced isoform STAT3β proteins. Functional domains: ND, NH2-terminal domain; CCD, coiled-coil domain; DBD, DNA binding domain; LD, linker domain; SH2, Src homology 2 domain; TAD, transactivation domain. Post-translational modifications: Ac, acetylation; Me, methylation; O-Glc-Nac, O-GlcNacylation; P, phosphorylation; Sumo, sumoylation.

Signal Transducer and Activator of Transcription (STAT) proteins are a seven-member family of cytoplasmic transcription factors that relay signals emanating from cell-surface cytokine and growth factor receptors to the nucleus [1,2]. STAT proteins control fundamental cellular processes, including survival, proliferation, differentiation, and immune responses [3]. It is now well-established that three of these members, STAT3 and the closely related STAT5A and STAT5B proteins (Figure 1) are also important effectors of cellular transformation. Aberrant STAT3, STAT5A, and STAT5B signaling have been described in different solid tumors such as prostate, breast, colon, gliomas, head and neck cancer, melanoma, and in hematopoietic malignancies [4,5,6,7] (see also [8] in this issue). Historically, persistent activation of these transcription factors was frequently found in many tumor cells as a consequence of deregulated tyrosine kinase activity. STAT5A/5B and/or STAT3 are downstream effectors of various tyrosine kinase oncogenes (TKOs) such as TEL-JAK2, JAK2^V617F^, SRC, TEL-ABL, BCR-ABL, TEL-SYK, NPM-ALK, TEL-PDGFR, and mutated forms of FLT3 and KIT receptors [9,10,11,12,13,14,15,16,17,18,19].

**Figure 2:**
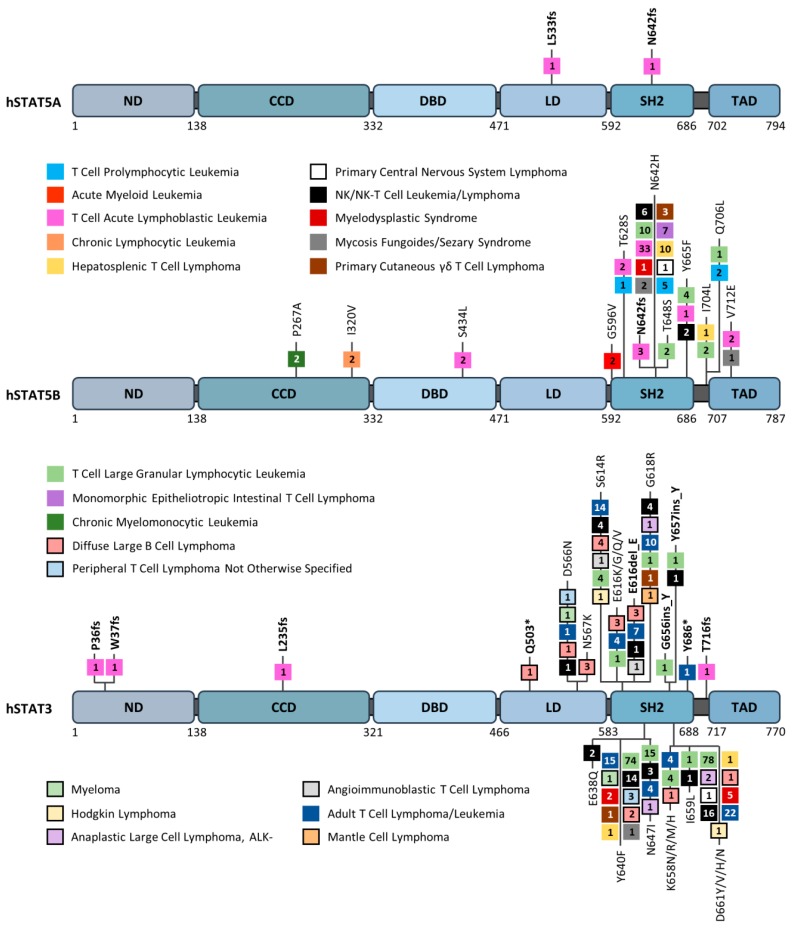
Map of somatic mutations detected in human STAT5A, STAT5B, and STAT3 in patients with hematologic cancers. Individual missense mutations found in at least two patients, as well as all reported nonsense (*) and frameshift (fs) mutations (bold), are depicted. The numbers in each box represent the number of cases reported for each mutation. Data were mined from the Catalogue of Somatic Mutations in Cancer (COSMIC) database. ND, NH2-terminal domain; CCD, coiled-coil domain; DBD, DNA binding domain; LD, linker domain; SH2, Src homology 2 domain; TAD, transactivation domain.

Inhibition of STAT3/STAT5 signaling has a negative impact on the transforming potential of these tyrosine kinases in vitro and in vivo. Evidence for a direct role of STAT3 or STAT5A/5B in cell transformation was provided by the use of constitutively active variants. These proteins, designated STAT3C, STAT51*6 or cS5^F^, are able to induce cell transformation in vitro and solid tumors or leukemias in vivo [20,21,22,23]. More recently, gain-of-function (GOF) mutations in STAT5B and STAT3 have been found in patients with leukemias and lymphomas (Figure 2) [24,25,26,27]. Occurring primarily within the SH2 domain, these mutations confer persistent and prolonged signaling and have been linked to poorer prognosis and relapse in patients [28,29,30]. Collectively, these data would undoubtedly define STAT3 and STAT5A/5B as important therapeutic targets in hematologic cancers. Nevertheless, STAT3 and STAT5 also behave as tumor suppressors in other tissues and regulate the antitumoral response of immune cells [31,32,33,34,35,36,37]. Thus, the respective and specific functions of STAT3 and STAT5, as well as their interactions in hematopoietic cancers, still need to be refined to develop therapeutic strategies that selectively block STAT3 and/or STAT5 activity in these diseases. In this review, we will first summarize the respective contribution of STAT3 and STAT5 in hematologic cancers as well as the canonical and non-canonical oncogenic properties of STAT3/STAT5. Finally, we will describe the different strategies used to target STAT3 or STAT5 and will discuss the potential future development of single or combined therapies to block STAT3 and/or STAT5A/5B activity/expression in hematologic cancers.

## 2. STAT3 and STAT5A/5B in Hematopoietic Cancers

### 2.1. STAT3/STAT5 in Hematopoietic Cancers: An Amazing 23-Year-Old Story

In 1996, pioneering works demonstrated that STAT3 and/or STAT5 are constitutively activated in leukemic cells from patients with acute myeloid leukemia (AML) or acute lymphoblastic leukemia (ALL) [38]. Surprising was the high constitutive STAT5 DNA binding activity detected in leukemic cells from Philadelphia chromosome-positive ALL (Ph^+^ ALL) patients. These original findings already pointed to STAT5 as a potential effector of the BCR-ABL tyrosine kinase fusion protein, the transforming agent in Ph^+^ ALL and chronic myeloid leukemia (CML), and were confirmed a few months later by other groups [15,16]. 23 years later with more than 4000 publications in the field, it is now clearly established that STAT3/STAT5A/STAT5B are essential and/or contribute to the development of hematopoietic malignancies, affecting both myeloid and lymphoid compartments (Table 1). Data also showed that deregulated STAT3 and STAT5 activity promotes drug resistance in leukemias/lymphomas/myelomas, highlighting the crucial interest to develop pharmacological molecules that selectively target STAT3 and/or STAT5 in hematologic cancers.

### 2.2. STAT3/5 in Myeloproliferative Neoplasms (MPNs)

Myeloproliferative neoplasms (MPNs) are hematologic diseases characterized by abnormal proliferation and accumulation of mature myeloid cells in the bone marrow and peripheral blood [39]. An increased risk of developing acute myeloid leukemia is also associated with MPNs. MPNs are classified as *BCR-ABL*-positive (or Ph^+^) and *BCR-ABL*-negative (Ph^−^) MPNs [40]. Both Ph^+^ and Ph^−^ MPNs are clonal disorders that result from the transformation of hematopoietic stem cells (HSCs). While the BCR-ABL fusion protein is the transforming agent in CML, driver mutations in *JAK2*, *CALR*, and *MPL* genes are variably present and are mostly mutually exclusive in Ph^−^MPNs, which include essential thrombocythemia (ET), polycythemia vera (PV), and primary myelofibrosis (MF) [41]. The JAK2 GOF mutation (JAK2^V617F^) has been identified in 95% to 97% of PV patients [42,43]. This mutation, located in the pseudokinase domain of the JAK2 protein, constitutively activates the kinase. JAK2, MPL, and CALR mutants have been functionally validated and are sufficient to induce MPNs in mice [41]. Systemic mastocytosis (SM), a subcategory of MPNs, is a heterogeneous clonal disorder characterized by an accumulation of mast cells in various organs [44]. The GOF mutation in KIT (KIT^D816V^) causing activation of the KIT receptor tyrosine kinase was found in 80–95% of patients with SM. Studies with transgenic mice suggested that this mutation alone is sufficient to cause SM [45]. The KIT^D816V^ mutant has also been detected in leukemic cells from AML patients [46]. The presence of KIT^D816V^ in AML is highly associated with co-existing SM [47]. Activation of STAT3 and/or STAT5 by BCR-ABL, JAK2^V617F^, and KIT^D816V^ has been abundantly documented in the literature. However, conflicting results (cell lines vs. primary cells and/or human vs. murine leukemic cells) have emerged from these studies. For instance, tyrosine phosphorylation of STAT3 (Y^705^) was observed in murine BCR-ABL^+^ cells but barely detected in human BCR-ABL^+^ cells [16,48]. Using *stat3*- or *stat5a/5b*-deficient mice, previous studies demonstrated that while STAT3 and STAT5 are required for the initial step of BCR-ABL-dependent cell transformation, only STAT5 is necessary for the maintenance of BCR-ABL-induced leukemia [49]. The effective role of STAT3 and STAT5 in maintenance, self-renewal, or transformation of normal HSCs might explain why both proteins are required in the BCR-ABL-dependent leukemia-initiating stem cell population [50,51,52,53]. More recently, dissection of the respective contributions of STAT5A and STAT5B in BCR-ABL-dependent transformation revealed that STAT5B, but not STAT5A, is a critical effector of BCR-ABL-driven leukemia development [54]. Besides survival- and growth-promoting effects, STAT5B facilitates leukemogenesis by suppressing IFN-α and IFN-γ signaling in these animal models. The development of tyrosine kinase inhibitors (TKIs) targeting BCR-ABL, such as imatinib mesylate (IM), has revolutionized the treatment of CML. However, imatinib mesylate (IM) is not totally curative and approximately 50% of patients remain therapy-free after IM discontinuation. The inability of IM to completely eradicate quiescent leukemic stem cells (LSCs) is probably responsible for the relapse of CML patients [55]. Moreover, the occurrence of BCR-ABL mutations in progressive or relapsed diseases promotes IM resistance of CML cells [56]. Studies indicated that high levels of phosphorylated STAT5 enhance the resistance of CML cells to TKIs but also triggers BCR-ABL mutations by inducing the production of reactive oxygen species (ROS) responsible for DNA damage [57,58,59]. Moreover, STAT5 was shown to play a key role in the maintenance of IM-resistant LSCs from CML patients [60,61].

The contribution of STAT3 in IM resistance was also demonstrated using an in vitro model of cocultures mimicking the bone marrow microenvironment of CML cells. In this model, bone marrow stromal cells stimulated the phosphorylation of STAT3 in CML cells in a BCR-ABL-independent manner and promoted the resistance of these leukemic cells to IM [62]. Interestingly, combined targeting of STAT3 and STAT5 has been proposed to overcome drug resistance in CML cells [63].

While STAT5 plays a critical role in JAK2^V617F^-driven mouse models of MPN, studies have shown that STAT3 is not required for myeloid expansion induced by this TKO [64,65,66,67]. Moreover, activation of STAT3 negatively regulates JAK2^V617F^-driven MPN in mice by enhancing thrombocytosis and shortening overall survival [67]. Further studies are therefore required to determine if STAT3 plays a pathogenic role in human MPNs.

STAT3, STAT5, and also STAT1 are activated by the KIT^D816V^ mutant in transformed mast cells, but only STAT5 seems to be transcriptionally active in these cells [68]. Phosphorylation of STAT5 has been detected in mast cells from SM patients. shRNA-mediated knockdown, dominant-negative mutant and pharmacological molecules targeting STAT5 all abrogate the growth of human neoplastic mast cells in vitro, indicating that STAT5 is a critical effector of KIT^D816V^ in human neoplastic mast cells [12].

Collectively, all data indicate that STAT5A/5B proteins are particularly relevant therapeutic targets in Ph^+^ MPN and Ph^−^ MPN and in the resistance to TKIs. Although the pathogenic role of STAT3 in human MPN still remains questionable, the contribution of STAT3 to TKI resistance elicited by the leukemic microenvironment would suggest that combination therapy or dual molecules targeting STAT3 and STAT5 might help to eradicate resistant leukemic cells in their “niche.”

### 2.3. STAT3/5 in Acute Myeloid Leukemia (AML)

AML is a heterogeneous clonal disorder characterized by immature myeloid cell proliferation and bone marrow failure. A two-hit model has been suggested as the probable mechanism in the pathogenesis of AML [69]. In this model, gene mutations that give a growth advantage and block normal hematopoietic differentiation are responsible for AML development. For instance, activating mutations in FLT3 (FMS-related tyrosine kinase 3) and KIT receptors promote proliferation and survival, while mutations affecting the transcription factor CEBPα inhibit myeloid differentiation. However, there are several other gene classes such as those involved in epigenetic regulation or metabolism that are mutated in AML [70].

Analyses of primary peripheral blood and bone marrow specimens have demonstrated constitutive activation of STAT3 and/or STAT5 in AML [6,37]. Importantly, constitutive activation of STAT3 and STAT5 has been linked with disease outcomes in AML. Bone marrow evaluation of AML patients revealed that the activation of STAT3 was significantly associated with poor overall survival and reduced progression-free survival [71]. In sharp contrast, the spliced STAT3β isoform was shown to have a suppressor function in AML [33]. A higher STAT3β/α mRNA ratio was found in AML cells and correlated with a favorable prognosis and increased overall survival. Stat3β expression in mouse models of AML resulted in decelerated disease progression and extended survival. It is, however, unclear whether tyrosine phosphorylation and dimerization are required for the tumor suppressor activity of STAT3β in these animal models. The contribution of STAT3 in AML may not only depend on the STAT3β/α ratio but also on the subtype of AML cells or acquired mutations in this disease. For instance, KIT^D816V^, which is frequently found in core-binding factor (CBF)-AML leukemias, stimulates autophagy through activation of STAT3 [72]. Inhibition of STAT3 blocked autophagy and reduced tumor growth in mouse xenograft models. Importantly, inhibition of STAT3 also stimulates the antitumoral immune response in animal models of AML, indicating that targeting STAT3 would not only block the growth and survival of AML but also AML-induced immune evasion [73,74].

Mutations in FLT3, either involving internal tandem duplications (FLT3-ITD) or point mutations in the activating loop of the tyrosine kinase domain (FLT3-KD), were observed in approximately 30% of AML patients and are associated with poor prognosis [75]. Although FLT3 mutants activate both STAT3 and STAT5 in cell lines, results showed that tyrosine-phosphorylated STAT5 is selectively associated with expression of FLT3-ITD in primary blasts from AML patients. Activation of STAT5 by FLT3-ITD is required to induce primary cell survival in vitro and leukemia in vivo [76,77,78,79,80]. Pharmacological inhibition of STAT5 also blocks FLT3-ITD-driven leukemias in mouse xenograft models [81]. FLT3-ITD promotes genomic instability by increasing ROS production via activation and association of STAT5 with the GTPase Rac1, which is an essential component of certain NADPH oxidases such as Nox2 [82]. Conversely, ROS production and p22phox, a membrane subunit of NADPH oxidase, are required for FLT3-ITD-induced STAT5 phosphorylation in the endoplasmic reticulum (ER) [83]. Importantly, recent works demonstrated that FLT3-ITD-independent activation of STAT5 induced by the leukemic microenvironment promotes resistance of FLT3-ITD^+^ AML cells to quizartinib, an FLT3 inhibitor that is now in phase 3 clinical trial [84].

Lastly, we should also mention the identification of a fusion between *STAT5B* and *Retinoic Acid Receptor (RAR)α* resulting from an interstitial deletion on chromosome 17 in acute promyelocytic leukemia (APL) [85]. The corresponding fusion protein enhances STAT3 signaling and blocks myeloid maturation by inhibiting RARα/retinoid X receptor (RXR)α transcriptional activity [86].

### 2.4. STAT3/5 in Acute Lymphoblastic Leukemia (ALL)

ALL is the most common form of cancer in children and predominantly arises from the transformation of B cell progenitors (80–85% of cases) [87]. Mouse studies suggest that STAT5 is functionally important in certain types of B-ALL [88]. Transgenic overexpression of a constitutively active STAT5A mutant (cS5^F^) cooperates with p53 deficiency to promote B-ALL in mice [89]. Genetic or pharmacological targeting of STAT5 suppresses human Ph+ ALL cell growth and leukemia development in mouse xenograft models [90]. Deregulation of precursor B cell antigen receptor (pre-BCR) signaling has been shown to be important in the development of B-ALL, and constitutive activation of STAT5B cooperates with defects in pre-BCR signaling components to initiate B-ALL [91]. Similarly, haploinsufficiency of B cell-specific transcription factors such as EBF1 or PAX5 synergizes with activated STAT5 in ALL [92]. Despite strong evidence for the oncogenic activity of STAT5 in TKO-driven B-ALL, the role of STAT5 appears to be context-dependent. For example, the deletion of STAT5 accelerates the development of B-ALL induced by c-myc in mouse models [93]. Activating mutations in *STAT5B* have been found in T-ALL [24,28]. The amino acid substitution N642H in the phosphotyrosine binding pocket of the SH2 domain promotes the constitutive activation of STAT5B and the capacity to induce T cell neoplasia in transgenic mice [29,30]. The role of STAT3 in ALL is poorly documented. However, data indicated that blockade of STAT3 signaling compromises the growth of B-ALL cells overexpressing the high mobility group A1 (HMGA1)-STAT3 pathway [94]. Unlike STAT5B, there are no recurrent STAT3 mutations detected in T-ALL and, in fact, only single frameshift mutations are reported (Figure 2).

### 2.5. STAT3/5 in T Cell Large Granular Lymphocytic (T-LGL) Leukemia

Activating mutations in the SH2 domain of STAT3 (Y640F, D661Y/V) and STAT5B (N642H) were also described in T-LGL leukemia which is a chronic lymphoproliferative disorder characterized by the expansion of some cytotoxic T cell or NK cell populations (Figure 2) [95,96,97]. *STAT3* mutations have been described in 30–40% of T-LGL leukemia patients while *STAT5B* mutations were found in rare but typical CD4^+^ T-LGL leukemia cases. However, *STAT5B* mutations were more frequently detected in patients with a severe clinical course. In all cases, mutations were shown to increase the transcriptional activity of both STAT3 and STAT5B proteins, but only the STAT5B^N642H^ mutation was demonstrated to drive T-LGL leukemias in mouse models [98,99].

### 2.6. STAT3/5 in Chronic Lymphocytic Leukemias (CLL)

CLL is characterized by the accumulation of mature clonal B cells in peripheral blood, bone marrow, and lymphoid tissues. These cells are characterized by an extended lifespan due to intrinsic defects in apoptosis [100]. Increasing STAT3 phosphorylation on S^727^ but not on Y^705^ is believed to be a hallmark of CLL progression [101]. Phosphorylation of S^727^ regulates the transcriptional activity of the STAT3 protein but it is also involved in the mitochondrial localization of STAT3 in primary cells from CLL patients [102]. Cytokines such as interleukin (IL)-15 secreted by the microenvironment contribute to the survival of CLL cells through JAK-mediated tyrosine phosphorylation of STAT5 [103].

### 2.7. STAT3/5 in Lymphomas

Lymphomas are cancers of the lymphatic system. They are divided into two categories: Hodgkin lymphoma (HL) and non-Hodgkin lymphoma (NHL). Data from the literature underscored the important contribution of STAT3 and STAT5 in the proliferation and/or survival of HL cells [104,105,106]. Most NHLs are B cell lymphomas. Diffuse large B cell lymphoma (DLBCL) is the most common subtype of NHL and consists of at least two phenotypic subtypes: the germinal center B cell-like (GCB-DLBCL) and the activated B cell-like (ABC-DLBCL) groups. High-level STAT3 expression and activation are preferentially detected in ABC-DLBCL, which is associated with poor outcomes [107,108]. Inhibition of STAT3 expression/activity in ABC-DLBCL cells abrogates lymphoma cell growth and triggers apoptosis. Moreover, STAT3 coordinates migration to facilitate the dissemination of DLBCL [109]. Among NHL subtypes, peripheral T cell lymphoma (PTCL) and natural killer (NK)/T cell lymphoma (NKTL) represent a heterogeneous group of diseases with varied clinical features, prognosis and response to treatment. PTCL has been categorized into several subtypes including PTCL-not otherwise specified (PTCL-NOS), angioimmunoblastic TCL (AITL), anaplastic large cell lymphoma (ALCL), and the predominant subsets of cutaneous TCL (CTCL) [110]. Deregulation of STAT3/STAT5 activity was shown to be important for CTCL pathogenesis and cancer progression [111]. ALCL can be divided into anaplastic lymphoma kinase (ALK) positive and ALK negative subgroups, based on ALK gene rearrangements. The nucleophosmin-anaplastic lymphoma kinase (NPM-ALK) fusion protein is the major oncogenic driver in ALK+ ALCL and it activates STAT3 and STAT5 [17,18]. While STAT3 is required for NPM-ALK-induced cell transformation and B cell lymphomagenesis, the contribution of STAT5 is still unclear [112]. Studies indicated that STAT5A, but not STAT5B, is epigenetically silenced in NPM-ALK tumors and behaves as a tumor suppressor when reactivated to suppress NPM-ALK expression [34]. Activating mutations of STAT3 and STAT5B have been found in NKTLs and γδ-T cell lymphomas [25,27]. Recurrent hot spot mutations Y640F and D661Y/V/H/N in the SH2 domain of STAT3 have been identified most commonly in T-LGL leukemia, NK, NK/T, and adult T cell leukemia/lymphoma (ATLL) patients, while the aggressive STAT5B^N642H^ mutation has been predominantly found in T-ALL and γδ-T cell lymphomas, such as hepatosplenic TCL, monomorphic epitheliotropic intestinal TCL, and primary CTCL (Figure 2) [113]. These mutations were shown to be associated with increased phosphorylated STAT3 and STAT5B proteins and to confer a growth advantage to transduced cell lines or normal NK cells [27]. Activating STAT3 mutations E616G and E616K in the SH2 domain found in NK/T cell lymphoma patients were shown to increase the phosphorylation and transcriptional activity of STAT3 [114]. Interestingly, programmed cell death-ligand 1 (PD-L1) was overexpressed in NK/T cells harboring hotspot STAT3 mutations, and overexpression of a STAT3^E616K^ or STAT3^E616G^ mutant was sufficient to enhance PD-L1 expression. These data are consistent with the role of STAT3 as an important regulator of tumor immune evasion [115]. In summary, STAT3 and STAT5B, but not STAT5A, are relevant therapeutic targets in the treatment of lymphomas. All recurrent STAT3 and STAT5B mutations in hematopoietic cancers are summarized in Figure 2.

### 2.8. STAT3/5 in Myelomas

Multiple myeloma (MM) is a B cell malignancy characterized by the proliferation of clonal plasma cells in the bone marrow accompanied by secretion of monoclonal immunoglobulin [116]. Constitutive tyrosine phosphorylation of STAT3 has been evidenced in MM cell lines and primary CD138^+^ cells from MM patients [117,118]. Patients with STAT3 activation were found to have significantly shorter progression-free and overall survival [119]. STAT3 activation has been reported to contribute to MM progression both directly, by upregulating survival and anti-apoptotic target genes, as well as indirectly by activating myeloid-derived suppressor-cells (MDSCs) in the bone marrow microenvironment, which facilitates tumor progression [120,121]. Myeloma cells rely on the pleiotropic cytokine IL-6, which activates the JAK/STAT3 pathway. IL-6 signaling is tightly regulated by tyrosine phosphatases SHP-1 and SHP-2, and suppressor of cytokine signaling 1 (SOCS1). The disruption of this negative feedback loop results in the constitutive activation of STAT3 [122]. Accordingly, *SHP-1* and *SOCS1* genes were found to be silenced by hypermethylation in MM patients [123]. In addition to the induction of anti-apoptotic gene expression in MM cells, STAT3 also regulates the expression of microRNA-21 with strong anti-apoptotic potential, suggesting that noncoding RNAs have an impact on the pathogenesis of human MM [124]. Moreover, the expression of five long noncoding RNAs (lncRNAs) was found to be regulated by STAT3 in MM cells [125]. However, the role of these lncRNAs in the progression of MM remains to be elucidated. The role of STAT5 seems to be marginal and restricted to immunoglobulin production in MM [126].

## 3. Canonical and Non-Canonical Roles of STAT3/STAT5 in Hematopoietic Cancers

**Figure 3:**
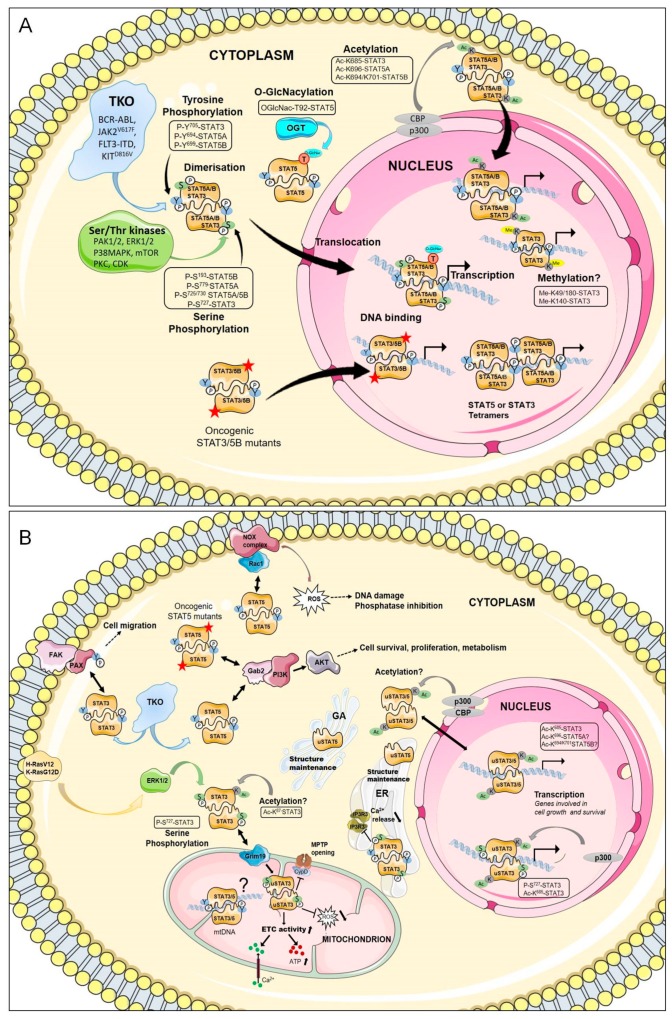
STAT3 and STAT5 signaling in hematologic malignancies. (**A**) Canonical function. In the canonical model, TKOs, or GOF mutations induce persistent activation (e.g., tyrosine phosphorylation) and nuclear translocation of STAT3 and STAT5, which then bind as dimers or tetramers to specific promoter sequences to regulate transcription of genes involved in cell growth, apoptosis, angiogenesis and immune response. Other post-translational modifications, as indicated, regulate the oncogenic activity of STAT3/STAT5. OGT (O-GlcNAc transferase). (**B**) Non-canonical function. uSTAT3 and uSTAT5 (non-tyrosine phosphorylated STAT3/5) proteins also have an active role in the nucleus, mitochondrion, ER (endoplasmic reticulum), GA (golgi apparatus), ETC (electron transport chain). Besides their transcriptional activity, P-Y-STAT3 and P-Y-STAT5 interact with different signaling pathways in the cytoplasm and ER.

It was presumed that most, if not all, of the oncogenic activities of STAT3/STAT5 are due to canonical functions. However, STAT3/5 proteins are also active through alternate non-canonical pathways impacting cell transformation. Post-translational modifications affecting canonical and non-canonical roles of STAT3 and/or STAT5 have been extensively reviewed in the literature. To be in line with this review, we will focus on canonical and non-canonical activities of STAT3/5 that have been described in cell transformation (Figure 3).

### 3.1. Canonical Function of STAT3/STAT5

In the canonical model, STAT3, STAT5A, and STAT5B are primarily activated by phosphorylation on tyrosine residues Y705, Y694, and Y699, respectively (Figure 1). In a physiological situation, STAT3 and STAT5 are transiently phosphorylated by JAK in response to cytokine receptor signaling, and contribute to hematopoietic cell proliferation and differentiation. In contrast, persistent tyrosine phosphorylation of STAT3/5 induced by TKOs promotes hematopoietic cell transformation [6,7]. Phosphorylation of additional tyrosine residues in STAT5A and STAT5B proteins in TKO-transformed cells has also been described in the literature, but the functional and physiological meaning of such phosphorylations remains unclear [127,128]. P-Y^705^-STAT3 (herein referred to in this review as P-Y-STAT3), P-Y^694^-STAT5A (P-Y-STAT5A), and P-Y^699^-STAT5B (P-Y-STAT5B) proteins dimerize through reciprocal interactions between the SH2 domain of one monomer and the phospho-tyrosine residue of the other. P-Y-STAT3, P-Y-STAT5A, and P-Y-STAT5B form homodimers but P-Y-STAT5A can also dimerize with P-Y-STAT5B. The formation of STAT5A/5B heterodimers is not only dependent on the relative abundance of each protein but can also be differentially affected by upstream signaling events. For instance, BCR-ABL is less efficient in inducing STAT5A/5B heterodimerization than IL-3 [128]. Heterodimers between P-Y-STAT3 and P-Y-STAT5A or P-Y-STAT5B have never been reported despite the capacity of some TKOs to simultaneously activate these proteins. After dimerization, STAT3, STAT5A, and STAT5B are translocated into the nucleus. Nuclear import is dependent on a nuclear localization signal present in the coiled-coil domain [129]. The nuclear import of STAT3/STAT5 occurs independently of their tyrosine phosphorylation and is mediated by the importin-α3/β1 system coupled to Ras-related nuclear (Ran) proteins bound to GDP or GTP [129]. Molecules other than importins and Ran also participate in the regulation of the nuclear translocation of STAT3/5. The small GTPase Rac1 and the GTPase-activating protein MgcRacGAP form a ternary complex with P-Y-STAT3 or P-Y-STAT5 to induce their translocation via the importin α/β pathway [130]. The MgcRacGAP/Rac1 complex also regulates the tyrosine phosphorylation of STAT3/5 induced by cytokines [131,132]. Interestingly, p21-activated kinases (PAK1 and PAK2), which are important effectors of Rac1, were shown to induce phosphorylation of the S779 residue and nuclear translocation of STAT5A in BCR-ABL-expressing cells [133]. In a similar vein, activation of focal adhesion kinase (FAK) by FLT3-ITD or KIT^D816V^ in AML cells was demonstrated to induce the nuclear translocation of STAT5 via the Rac1/PAK1 pathway [134]. In sharp contrast, several reports indicated that P-Y-STAT5 is abundantly detected in the cytoplasm of BCR-ABL^+^ cells and that Src kinases might be responsible for the cytoplasmic retention of P-Y-STAT5A [135,136]. The reasons for these apparent controversial data remain unclear but might be related to the cellular context.

Tyrosine phosphorylated STAT3/STAT5 dimers bind to specific DNA elements found in promoters, enhancers, and the first intron of target genes. These binding sites are characterized by clusters of conserved motifs with an interferon gamma-activated site (GAS)-like core sequence (TTCT/CNA/GGAA). STAT5 was shown to bind as a tetramer on adjacent GAS sequences with high or weak affinity in target gene promoters [137,138]. The tetramerization domain located in the NH2-terminal region of STAT5 was found to promote constitutively active, STAT5A mutant (cS5^F^)-induced leukemia in mice [23]. The NH2-terminal domain of STAT3 is also required for oligomerization and IL-6-dependent transcriptional regulation [139]. This domain is involved in STAT3-mediated survival of solid tumor cells but its role in hematologic cancers is currently unknown [140].

TKO-mediated activation of STAT3 and/or STAT5 regulates expression of common as well as specific genes involved in hematopoietic cell survival, proliferation, metabolism, hypoxia, autophagy, migration and tumor immune evasion. BCL2-family members, cell cycle-regulated genes, proto-oncogenes such as PIM1, c-MYC, BCL6, and genes involved in cytokine receptor signaling or immune response are often targets of STAT3/STAT5-mediated transcription in hematopoietic cancers [141]. Transcription of these genes is also dependent on P-Y-STAT3/5 levels in the nucleus. For instance, previous works demonstrated that nuclear P-Y-STAT5 proteins at low, intermediate, and high levels differentially affect self-renewal, proliferation, and differentiation of leukemic stem cells by regulating the expression of distinct sets of genes [142]. Accordingly, P-Y-STAT5 regulates D-type cyclins and c-MYC expression at intermediate levels to promote proliferation but upregulates expression of the cyclin-dependent kinase (CDK) inhibitor p21waf1 at high levels to induce growth arrest and differentiation. Similarly, while constitutively active STAT3 was shown to protect CLL cells from apoptosis, at high levels it induced apoptosis and caspase-3 expression [143]. These data emphasize that increasing nuclear levels of P-Y-STAT3/5 might confer a growth disadvantage to leukemic cells [143,144]. Protein–protein interactions also drive the regulation of STAT3/STAT5-dependent gene expression. STAT3 and STAT5 interact with many transcription factors, co-factors, and/or chromatin remodeling proteins such as enhancer of zeste homolog 2 (EZH2), CREB-binding protein (CBP)/p300, ten-eleven translocation 2 (TET2), and DNA methyltransferase 1 (DNMT1), which largely impact hematopoiesis or leukemogenesis. Blocking these proteins also interferes with STAT3/STAT5-mediated transcription and cancer cell growth. Most of these interacting partners were exhaustively described in a recent review and will not be discussed here [141]. In addition, serine phosphorylation plays an important role in STAT3/5-dependent cell transformation. Constitutive phosphorylation of S727, which is located in the COOH-terminal transactivation domain of STAT3 (Figure 1), was shown to increase the transcriptional activity of this protein in CLL cells [101]. The oncogenic activity of STAT5A is highly dependent on S725 and S779 phosphorylation, and both residues (S726 and S780 in humans) were found to be constitutively phosphorylated in AML, ALL, and CML cells [145]. Importantly, PAK-dependent S779 phosphorylation of STAT5A is required for BCR-ABL-induced leukemogenesis but is not affected by BCR-ABL tyrosine kinase inhibitor treatment, indicating that oncogenic activation of STAT5A is mediated by independent pathways in BCR-ABL-expressing cells [133]. Phosphorylation of S779/780 on STAT5A also promotes the expansion and transformation of human hematopoietic stem/progenitor cells (HSCs/HPCs) [53]. Constitutive phosphorylation of S193 on STAT5B has been detected in various lymphoid tumor cell lines as well as in primary cells from leukemia or lymphoma patients [146]. Phosphorylation of S193 was found to be sensitive to inhibitors of mammalian targets of rapamycin (mTOR) and to play an important role in the DNA binding and transcriptional activity of STAT5B. Other post-translational modifications such as glycosylation affect the oncogenic functions of STAT5. O-GlcNAcylation at threonine 92 in STAT5A and STAT5B was shown to regulate tyrosine phosphorylation and transcriptional activity of oncogenic STAT5 in leukemic cells [147].

STAT3 and STAT5 also undergo acetylation on multiple lysine residues by the CBP/p300 histone acetyltransferase [148]. Acetylation of K685 located in the SH2 domain of STAT3 can enhance transcriptional activity by increasing dimer stability. Mutation of K685 affects dimerization but not tyrosine or serine phosphorylation of STAT3, and acetylation of STAT3 can occur in the absence of tyrosine phosphorylation [149,150]. AcK^685^-STAT3 also recruits DNMT1 to induce the epigenetic silencing of tumor suppressor genes [151]. Acetylation of STAT5A and STAT5B at K696 and K694/K701 residues, respectively, is also required for STAT5 dimerization [152]. The contribution of acetylation in the canonical functions of oncogenic STAT3/5 on one hand, and in hematopoietic cancers, on the other hand, remains very unclear. Previous work indicated that acetylation of K685 does not play an essential role in the expression of the great majority of P-Y-STAT3-dependent genes, suggesting that acetylation might be more important for the non-canonical functions of STAT3/5 [153].

Methylation of nuclear STAT3 on K140 or K49/K180 by the histone-modifying enzymes SET domain-containing lysine methyltransferase 9 (SET9) or EZH2, respectively, have been reported to mediate opposing effects on STAT3-dependent transcriptional activity [154,155,156]. STAT5 also interacts with EZH2 in B cells to repress the Igκ locus [157]. The methylation status of STAT3 and STAT5 in hematopoietic cancers has yet to be investigated. Similarly, other post-translational modifications such as oxidation, glutathionylation, or sumoylation were shown to negatively regulate STAT3/5 activity, but their impact on STAT3/5-driven hematologic malignancies are still unknown [158,159,160,161,162].

The concomitant activation of STAT3/5 by TKOs is probably the most intriguing event in leukemogenesis as both proteins in cancer cells have compensatory and/or opposing effects on gene expression and cell fate [163]. For instance, the competitive binding of STAT3 and STAT5 to the regulatory loci of BCL6 and IL-17 has been shown to modify gene expression and cell phenotype [164,165]. The differential recruitment of co-activators and/or co-repressors might explain these opposing effects on gene expression. Activation status of both STAT5 and STAT3 might, therefore, provide important diagnostic and prognostic information in hematologic cancers. Notably, JAK2^V617F^ activates both STAT3 and STAT5, but only STAT3 negatively regulates JAK2^V617F^-dependent MPN development [66,67].

### 3.2. Non-Canonical Functions of STAT3/STAT5

Subcellular localization of P-Y-STAT3/5 is not restricted to the nucleus and previously published data highlighted important functions of P-Y-STAT3/5 in the cytoplasm and/or other subcellular organelles of cancer cells. Cytoplasmic localization of P-Y-STAT5 was abundantly found in leukemic cells expressing BCR-ABL, JAK2^V617F^, or KIT^D816V^. Here, it was shown to interact with the scaffolding adapter Gab2 to favor the activation of the phosphatidylinositol-3-kinase (PI3K)/AKT pathway and leukemic cell survival [12,135,166]. The deletion of Gab2 attenuated the transforming potential of an oncogenic STAT5 mutant in mouse models [167]. Interaction of P-Y-STAT5 with Rac1, which is also an important component of certain membrane-bound NADPH-oxidase (NOX) enzymatic complexes, promotes ROS production in FLT3-ITD-expressing AML cells thereby increasing cell growth concomitantly with DNA damage [82]. BCR-ABL- and JAK2^V617F^-induced ROS generation is mediated by P-Y-STAT5, and binding of P-Y-STAT5 to the NOX2 complex in BCR-ABL^+^ cells also requires active Rac1 (unpublished data) [58,59,168]. P-Y-STAT3 has been detected in focal adhesions of cancer cells where it interacts with phosphorylated paxillin and FAK, thereby regulating cell migration [169]. Constitutively active STAT3 also controls Ca2^+^ release in the ER by interacting with inositol 1,4,5-triphosphate receptors (IP3R3), facilitating its proteasomal degradation [170]. Accordingly, the binding of STAT3 to IP3R3 protects cells from oxidative/ER stress and apoptosis. Finally, the mitochondrial localization and the potential role of P-Y-STAT3 and P-Y-STAT5 in regulating the mitochondrial genome have also been reported [171,172,173]. Collectively, these data suggest that P-Y-STAT3/5 distribution in subcellular organelles or cytoplasmic structures may directly impact cancer cell growth and survival independently of their transcriptional activity in the nucleus. It would then be relevant to explore in further detail the subcellular localization and function of P-Y-STAT3/5 in hematologic malignancies.

Constitutive phosphorylation of STAT3 on residue S727, but not on Y705, plays a role in the pathogenesis of CLL by regulating STAT3-dependent expression of genes associated with cell growth and survival [101]. DNA binding and transcriptional activity of P-S^727^-STAT3 probably require acetylation of K685 because (1) persistent acetylation of K685 on STAT3 has been observed in CLL cells, (2) acetylation of K685 promotes dimer formation, DNA binding and activation of transcription, and (3) K685 acetylation provides CLL cells with survival advantages [174]. In addition to its nuclear localization, P-S^727^-STAT3 was also found in mitochondria where it contributes to the viability of CLL cells and protects against oxidative stress [102]. The oncogenic activity of mitochondrial STAT3 was first demonstrated in H-Ras^V12^-transformed cells where it promotes anchorage-independent cell growth and tumor induction in mice [175]. Phosphorylation of S727 is carried out by the MAPK/ERK kinase (MEK)/Extracellular signal-regulated kinases (ERK) pathway and is necessary for Ras-mediated cell transformation [176]. Mitochondrial P-S^727^-STAT3 also supports K-Ras^G12D^-driven hematologic neoplasms [177]. Retinoid interferon-induced cell mortality (GRIM)19, a component of the electron transport chain (ETC) complex 1 and binding partner of STAT3, was shown to mediate STAT3 uptake into mitochondria [178]. However, other proteins such as the chaperone TOM20 have also been suggested as mitochondrial carriers of STAT3 [179]. Phosphorylation of S727 is important for mitochondrial STAT3 to upregulate ETC activity and ATP production, and mutation of S727 decreases the mitochondrial translocation of STAT3 [178]. In addition, CBP-dependent acetylation of K87 was also found to promote the mitochondrial localization of STAT3 [180]. Hematopoietic cell-targeted deletion of the *stat3* gene demonstrated the critical role of mitochondrial STAT3 in HSC/HPC function. *stat3*^−/−^ mice have a blood phenotype with similarities to human diseases of myelodysplastic syndrome (MDS) and MPN, supporting, as mentioned above, a negative regulatory role of STAT3 in MPN development [181]. These data also suggest that targeting mitochondrial STAT3 in K-Ras-induced hematopoietic malignancies might have adverse effects on normal HSCs. In addition to mitochondrial functions, non-tyrosine phosphorylated STAT3 and STAT5 proteins (uSTAT3/5) also have important roles in the nucleus, ER, and Golgi apparatus (GA). The knockdown of STAT5A/5B in human pulmonary arterial endothelial and smooth muscle cells resulted in a loss of uSTAT5A and uSTAT5B associated with the GA, leading to a dramatic destabilization of the ER and GA. This was accompanied by the activation of ER stress pathways, indicating a potential role of uSTAT5 in maintaining the structure and function of these organelles [182]. Inhibition of uSTAT5 activity/expression induced ROS production and apoptosis in pre-B leukemic cells, indicating that uSTAT5 may also provide protection against oxidative stress [183] (unpublished data).

uSTAT3/5 are also involved in the regulation of transcription and chromatin remodeling as preformed anti-parallel dimers [129,141]. The NH2-terminal domain mediates the dimerization of uSTAT3 and is essential for its nuclear accumulation, DNA binding, chromatin remodeling, and regulation of gene expression [184,185]. Acetylation of K685 was shown to be crucial for uSTAT3 to form stable dimers and regulate gene transcription [149]. Accordingly, the majority of uSTAT3-mediated gene expression depends on the ability of K685 to become acetylated [153]. Interestingly, nuclear uSTAT3 regulates the expression of well-known genes in cancer, suggesting that uSTAT3 might contribute to oncogenesis [186]. The role of uSTAT5 is less documented but data from the literature indicates that nuclear uSTAT5 behaves as a partial antagonist of P-Y-STAT5 and acts as a repressor to maintain self-renewal of hematopoietic cells and to block differentiation [187]. The role of uSTAT3/5 in the initiation, emergence and/or progression of hematologic cancers has yet to be determined. However, previous studies suggested that uSTAT5 provides preferential and critical cell survival signals in lymphoid tumor cells, indicating that uSTAT3/5 should also be considered as therapeutic targets in certain hematologic malignancies [188]. Our understanding of the molecular mechanisms involved in non-canonical functions of STAT3/5 is still incomplete. Most of these activities contribute to the known roles of STAT3/5 in hematopoiesis and hematopoietic neoplasms, and this knowledge complicates the already difficult task of targeting STAT3/5 for therapeutic purposes.

## 4. Pharmacological Inhibitors of STAT3 and STAT5

### 4.1. Introduction

Modalities for targeting STAT3 and STAT5 in hematologic cancers can be classified into direct and indirect approaches. Compounds directly targeting STAT3 or STAT5 canonical functions may either inhibit dimerization, DNA binding, or transcriptional activity. Indirect approaches include preventing ligands binding to growth factor or cytokine receptors, inhibiting upstream tyrosine kinases such as TKOs, targeting the nucleocytoplasmic shuttling of STAT3/5, or activating negative regulators of STAT3/5 such as the tyrosine phosphatases SOCS or PIAS [189,190,191]. Both direct or indirect approaches might also be applied to non-canonical functions. Molecules targeting STAT3/5 protein expression/stability or disrupting interactions with partners that play critical roles in oncogenic STAT3/5 activity should also be considered.

Historically, abnormal activation of STAT3 in solid tumors was first appreciated before STAT5 was implicated, and as such, various therapeutics to target STAT3 were first developed based on one or more of these strategies described above. Pioneering works using phospho-Y-peptides to compete with P-Y^705^ for binding to the SH2 domain of STAT3 and peptidomimetic derivatives provided the proof of concept that disrupting the P-Y^705^/SH2 domain interaction could be an efficient strategy to block oncogenic STAT3 signaling [192,193]. However, peptides and peptidomimetics have certain limitations in line with their in vivo instability and poor membrane permeability [194]. Nevertheless, these limitations provided the necessary impetus in many programs to design small molecules with greater and more specific inhibitory effects. Blocking DNA binding using decoy oligonucleotides (Duplex ODN), G-quartet oligonucleotides (GQ-ODN), or DBD peptide aptamers was another strategy to suppress canonical functions of STAT3 and STAT5. Treatment with STAT3 or STAT5 ODNs inhibits cell growth and/or induces apoptosis by preventing nuclear translocation of STAT3/5 in cancer cells [195]. RNA interference (RNAi) or antisense oligonucleotides (ASO) were also employed to target STAT3/5 mRNA in leukemia or lymphoma cells [195]. Some of these tools have been promising in modulating STAT3/5 signaling and inhibiting tumor cell growth. However, the therapeutic success of these different approaches relies on the effective entry and stability of the oligonucleotides in the cells and therefore requires chemical modifications for this purpose. Finally, cell-based screening with chemical compound libraries has allowed the identification of natural, synthetic, or clinically used molecules that inhibit STAT3/5 transcriptional activity [196,197]. However, hits derived from these assays have indirect effects that are challenging to determine. Due to an ever-growing list of STAT3/5 inhibitors and reviews in the field, we will focus on those that have been tested in hematopoietic cancers. We will discuss the limitations of STAT3/5 inhibitors in the treatment of these diseases but also promising outcomes when combined with other pharmacological compounds.

### 4.2. Indirect Inhibitors of STAT3 and STAT5 Signaling in Hematopoietic Cancers

#### 4.2.1. Targeting Upstream Tyrosine Kinases

Most agents that are described as indirect STAT3/5 inhibitors actually target upstream kinases such as JAK, Src, BCR-ABL, FLT3, or KIT receptors. Activation of STAT3 and/or STAT5 is dependent on the leukemic cell type in which the kinase is active. The development of IM and related BCR-ABL kinase inhibitors such as nilotinib, dasatinib, bosutinib, and ponatinib has made a major breakthrough in targeted cancer therapy, CML and Ph^+^ALL treatment [198,199,200,201,202,203,204]. IM leads to complete inhibition of BCR-ABL-dependent STAT5 activation and this is likely an important part of the effectiveness of this molecule [49,90]. However, some patients cannot tolerate the side effects of IM and related TKIs. In addition, the development of resistance to TKIs is a significant clinical problem, and is due, in part, to acquired point mutations in BCR-ABL such as T315I [205]. Although second- and third-generation TKIs were found to be effective against some BCR-ABL mutants, TKIs alone do not eradicate LSCs and de novo resistance of CML cells [55,56,62]. Targeting the kinase activity of FLT3 mutants has also been adopted to inhibit aberrant signaling in AML [206]. Among the agents that have been or are being evaluated in preclinical studies and/or clinical trials are multi-kinase inhibitors midostaurin (PKC412), sorafenib (BAY 43-9006), lestaurtinib (CEP701), KW-2449 (which also targets T315I-mutated BCR-ABL), sunitinib (SU11248), tandutinib (MLN518), and quizartinib (AC220), as well as compounds more specific for FLT3 mutants, including crenolanib (CP-868596) and gilteritinib (ASP2215) (Table 2) [207,208,209,210,211,212,213,214,215,216,217,218,219]. Few of them were approved for the treatment of FLT3-mutated AML as a single-agent or in combination with other therapeutic drugs. Although the effectiveness of these inhibitors was shown in preclinical studies, mixed results have been observed in clinical trials. Clinical activity of some of these molecules was evidenced in patients with FLT3-mutated AML but was often transient and relapse eventually occurred [206]. Secondary mutations in FLT3 and/or mutations associated with epigenetic regulators or transcription factors are responsible for the loss of therapeutic response to FLT3 inhibitors [206,220]. In addition, the bone marrow microenvironment provides protection against FLT3-TKIs [221]. Midostaurin, crenolanib, and tandutinib were also employed to inhibit KIT^D816V^ activity in mast cells from SM patients, while lestaurtinib was tested in two phase 2 trials for MPN treatment (Table 2) [222,223,224,225,226]. Other potent and selective KIT^D816V^ inhibitors including avapritinib (BLU-285) and DCC-2618 have entered clinical trials with promising results for the treatment of SM [222].

A breakthrough in understanding myeloproliferative diseases occurred after the discovery of GOF mutations in JAK2, leading to the development of small-molecule inhibitors of JAK2 for the treatment of MPNs [227]. In clinical trials, responses obtained with JAK inhibitors are independent of the driver mutations. However, treatment with JAK inhibitors was shown to have some limitations, partly because the targeted pathway is required for normal hematopoiesis and because specific inhibitors targeting the JAK2^V617F^ mutant are yet to be developed. Consequently, JAK2 inhibitors have been disappointing in their ability to induce molecular remissions in MPN patients, indicating that JAK2 inhibitors do not preferentially target MPN cells over normal cells [228]. In addition, some of the JAK inhibitors that entered clinical trials were discontinued due to their adverse effects. A crystal structure and biochemical properties of the pseudokinase domain of JAK2 will certainly assist in developing JAK2^V617F^-specific inhibitors in the future. Selective JAK and pan-JAK inhibitors that variably affect P-Y-STAT3/5 levels in hematologic neoplasms are presented in Table 2. Ruxolitinib is the first clinically-approved JAK1/2 inhibitor for PV and MF treatment and is also in clinical trials either alone or in combination with other pharmacological agents or TKIs for HL, MM, or CML treatment (Table 2) [227,228,229,230,231,232]. Ruxolitinib and most of the JAK inhibitors that are in clinical trials are type I inhibitors, which means that they block the ATP-binding site of JAKs under the active conformation of the kinase domain [227,233,234,235,236,237]. Type II inhibitors bind to the ATP-binding pocket of the JAK2 kinase domain in the inactive conformation, while allosteric inhibitors interact with other sites in the JAK2 protein [227]. Importantly, JAK2 target inhibition in MPN can be improved with the type II inhibitor NVP-CHZ868 offering increased therapeutic efficacy [238]. NVP-CHZ868 was also shown to act synergistically with dexamethasone in suppressing the growth and survival of human B-ALL cells in PDX models [239]. JAK2-specific inhibitors such as pacritinib are in the final stages of clinical trials for primary and secondary MF, and display increased potency compared to currently available JAK inhibitors [233]. Gandotinib, which is in a phase 2 study for JAK2^V617F^-mutated MPN treatment, showed an increased potency for the JAK2^V617F^ mutant [240]. Another example of a JAK2 inhibitor, fedratinib, which was previously burdened with a clinical hold in 2013, was recently FDA-approved for the treatment of MPN patients who have failed therapy with ruxolitinib [241]. Fedratinib also blocks the growth of HL and Mediastinal Large B-cell Lymphoma (MLBCL) in vitro and in vivo as demonstrated in preclinical studies [242]. Many JAK inhibitors with different selectivity and/or mechanisms of action have been tested in leukemias, lymphomas and MM (Table 2) [243,244,245,246,247,248,249,250,251,252,253,254,255,256,257,258,259,260,261,262,263,264,265,266,267,268]. For instance, AZD1480, INCB20, INCB16562, NS-018 and momelotinib (CYT-387) showed promising in vitro and/or in vivo efficacy against MM cells (Table 2) [246,247,248,251,261]. In all cases, P-Y-STAT3 was markedly reduced in MM cells treated with these inhibitors. Collectively, these data strongly support that JAK inhibition has significant potential as a therapeutic strategy in MM.

#### 4.2.2. Natural and Synthetic Molecules

Historically, natural compounds have been successfully used in the management of various human diseases. Natural products may also serve as a basis for the synthesis of derivatives aiming to increase their efficacy. There are several compounds that are known to exert anti-tumor effects through their indirect or direct action on STAT3 and/or STAT5 signaling. These natural molecules have a low toxicity profile and can act synergistically with other pharmacological agents to reverse chemoresistance. A number of plant-derived molecules such as avicin D, curcubitacin I, butein, honokiol, capsaicin, celestrol, and piperlongumine have been reported to inhibit growth or survival of leukemia, lymphoma, or myeloma cells in preclinical studies (Table 2) [269,270,271,272,273,274,275,276,277]. However, the mechanistic basis of their effects on STAT3/STAT5 signaling is still unknown. Inhibition of upstream kinases JAK1/2 and Src, and upregulation and/or activation of SHP1 or other protein tyrosine phosphatases appear to be a common feature of these compounds. Curcumin, a naturally derived phytochemical from plants such as turmeric (*Curcuma longa*), has been extensively investigated for its anti-tumor effects [278]. Curcumin was shown to block STAT3 and/or STAT5 phosphorylation in leukemia, lymphoma, and myeloma cells (Table 2) [279,280,281,282,283]. Although the administration of curcumin has been shown to be safe in humans, its clinical utility is somewhat limited due to the poor bioavailability and target selectivity. Therefore, efforts were made to design and synthesize novel curcumin analogs. FLLL32, one of these analogs, was shown to inhibit P-Y-STAT3 and growth of MM cells with greater efficacy but, again, target selectivity and mechanisms of action remained poorly defined [284]. The synthetic triterpenoid, CDDO-Imidazolide, which acts as an anti-inflammatory and anti-cancer drug, was demonstrated to suppress STAT3 and STAT5 phosphorylation and to induce apoptosis in MM cells [285]. EC804, a synthetic derivative of indirubin, an active component in a traditional Chinese medicine formulation, was reported to inhibit STAT3 and/or STAT5 phosphorylation as well as the growth of sensitive or TKI-resistant CML and AML cells [286,287]. This compound also blocks STAT3 activity in solid tumors [288]. Natural and synthetic compounds such as sulforaphane and the BET inhibitor JQ1 were found to inhibit STAT5-mediated transcription in CML and T-ALL cells, probably via epigenetic mechanisms [289,290]. Naphthoquinone (NPQ)-based derivatives could be also mentioned as indirect inhibitors of STAT5 probably through their multikinase modulatory effects in leukemias [291]. Research in our laboratory is focused on the synthesis and development of small-molecule inhibitors of STAT5. We recently identified 17f as a compound that selectively inhibits STAT5 phosphorylation and expression in AML and CML cells [292]. Moreover, we found that 17f overcomes the resistance of CML and AML cells to IM and Ara-C, a conventional therapeutic agent used in AML treatment [293]. We also found that 17f, when associated with IM or Ara-C, inhibits expression of STAT5B but not STAT5A in resistant CML and AML cells via translational or post-translational mechanisms. The mechanistic basis of this inhibitory effect is currently under investigation.

#### 4.2.3. Drug Repositioning

Cell-based assays for high-throughput screening were employed to identify compounds that specifically block the transcriptional activity of STAT3/5 [196,197]. This type of strategy utilizes cells that are stably transfected with a construct containing the luciferase reporter gene under the control of a specific and high-affinity STAT3 or STAT5 responsive promoter. Chemical libraries of compounds biased toward bioactives and drugs known to be safe in humans were used in the screening. Using this approach, nifuroxazide, niclosamide, and pyrimethamine were identified as specific inhibitors of STAT3, while pimozide was found to inhibit STAT5 activity (Table 2). Nifuroxazide, an antidiarrheic agent, was shown to decrease STAT3 tyrosine phosphorylation, most probably via inhibition of JAK kinase activity, and to reduce the viability of MM cells [294]. Niclosamide, an antiparasitic drug, blocks P-Y-STAT3 and myeloma cell growth via unknown mechanisms. Niclosamide lacks selectivity because it also inhibits NFkB activity in MM cells [295]. The antiparasitic and antifolate drug pyrimethamine also displays significant activity in vitro against MM cell lines harboring P-Y-STAT3 [196]. Pyrimethamine inhibited P-Y-STAT3 and transcriptional activity without affecting its upstream kinase JAK2. Three-dimensional modeling studies indicated that pyrimethamine binds to the SH2 domain, suggesting that it might be a direct inhibitor of STAT3, but this interaction needs to be biophysically demonstrated. Pyrimethamine was also found to be a potent inducer of apoptosis in AML cells [296]. However, it is still unclear whether the antitumor activity of pyrimethamine is due to its inhibitory effect on STAT3 and/or on folic acid metabolism [297]. Pyrimethamine is currently in a phase 1 clinical trial for the treatment of high-risk MDS and in phase ½ trials for CLL and small lymphocytic lymphoma (SLL) treatment.

Pimozide was identified by high-throughput drug screening as a potent inhibitor of STAT5. Pimozide was shown to inhibit P-Y-STAT5 and survival of CML and MPN cells without affecting the kinase activity of BCR-ABL, JAK2 or Src [298,299]. Pimozide shows synergistic effects with IM/nilotinib in killing CML cells and overcomes TKI resistance in BCR-ABL^T315I^ mutant cells [298]. The effects of pimozide are not limited to CML and MPN cells, and the efficacy of this drug was also demonstrated in AML. Pimozide can also inhibit P-Y-STAT5 and STAT5-dependent gene expression in AML cells expressing FLT3-ITD, and it acts synergistically with FLT3 inhibitors to induce apoptosis in these leukemic cells [79]. The mechanisms involved in pimozide-mediated inhibition of P-Y-STAT5 is currently not known. Pimozide also inhibits P-Y-STAT3 in myeloma cells indicating that it is not a selective STAT5 inhibitor [196]. Antidiabetic drugs such as pioglitazone and rosiglitazone were shown to have antileukemic activity [300]. Both synthetic compounds belong to the thiazolidinedione (TZD) class of ligands that bind to the nuclear receptor PPARγ. Activation of PPARγ by pioglitazone not only inhibits the growth of CML cells but also reduces the expression of *STAT5* genes [60]. Quiescent CML stem cells, which are known to be resistant to TKI treatment, are highly sensitive to pioglitazone when combined with IM. This suggests that besides phosphorylation, targeting STAT5 expression might be important for eradicating resistant CML stem cells. Whether PPARγ directly regulates STAT5A and STAT5B gene promoter activity remains to be investigated. Pioglitazone combined with IM are now in a phase 2 trial to evaluate the impact of this combination therapy on CML residual disease [301]. Activation of PPARγ was also shown to induce apoptosis in Ph^+^ ALL cells [302]. Finally, PPARγ agonists were found to inhibit the transcriptional activity of STAT3 in MM cells. In that case, it has been proposed that PPARγ and STAT3 may compete for binding to nuclear co-factors [303].

**Table 2:** Indirect inhibitors of STAT3 and STAT5 that have been tested in hematologic cancers.

Drug	Target(s) and Mode of Inhibition	Hematologic Malignancy	Stage of Clinical Development	References
**Upstream Kinases**				
**Atiprimod**	JAK2/JAK3 Inhibits kinase activity	AML JAK2^V617F^-MPN	Preclinical (cell lines, primary cells) Preclinical (cell lines, primary cells)	[249,250]
**AZ960**	JAK2/JAK3 Type I inhibitor	T cell leukemia/ lymphoma	Preclinical (cell lines)	[251]
**AZD1480**	JAK2/JAK1/Aurora/FGFR1/FLT4 Type I inhibitor	MPN B-ALL Lymphomas MM	Phase 1 (terminated) Preclinical (xenografts) Preclinical (cell lines) Preclinical (cell lines, xenografts)	[243,244,245,246]
**Bosutinib** **(SKI-606)**	BCR-ABL/Src Type I inhibitor	CML	Market authorization (Bosulif^®^, Pfizer)	[201,202]
**Crenolanib** **(CP-868596)**	FLT3/PDGFR/KIT Type I inhibitor	FLT3-mutated AML SM, AML	Phase 2 Phase 3 (CT) Preclinical (cell lines, primary cells)	[218,223]
**Dasatinib** **(BMS-354825)**	BCR-ABL/Src. Inhibits kinase activity Type I inhibitor	CML, Ph^+^ ALL	Market authorization (Sprycel^®^, BMS)	[200]
**Fedratinib (TG101348)**	JAK2/FLT3/BRD4 Type I inhibitor	MPN HL, MLBCL	Market authorization (Inrebic^®^, Celgene) Preclinical (cell lines, xenografts)	[241,242]
**Gandotinib (LY2784544)**	JAK2/JAK1/JAK3 Type I inhibitor	JAK2^V617F^-MPN	Phase 2	[240]
**Gilteritinib** **(ASP2215)**	FLT3/AXL Type I inhibitor	FLT3-mutated AML	Market authorization (Xospata^®^, Astellas)	[219]
**Imatinib** **(STI-571)**	BCR-ABL. Type II inhibitor	CML, Ph^+^ALL	Market authorization (Glivec^®^, Novartis)	[198,199]
**INCB20**	Pan JAK	MM	Preclinical (cell lines, primary cells, mouse models)	[247]
**INCB16562**	JAK1/JAK2. Type I inhibitor	MM	Preclinical (cell lines, primary cells)	[248]
**Itacitinib** **(INCB-039110)**	JAK1 Type I inhibitor	MF B-cell lymphoma	Phase 2 Phase 1/2	[252,253]
**KW-2449**	FLT3/BCR-ABL/ BCR-ABL315I/Aurora Inhibits FLT3 and BCR-ABL phosphorylation	CML, AML MDS, AML, ALL, CML	Preclinical (cell lines, primary cells, xenografts) Phase 1	[212,213]
**Lestaurtinib** **(CEP-701)**	JAK2/FLT3/TrkA/ Aurora kinase Type I inhibitor	AML MF PV, ET	Phase 2 Phase 2 Phase 2	[211,225,226]
**LS104** **(AG490 analog)**	JAK2/BCR-ABL Allosteric inhibitor	JAK2^V617F^-MPN AML Ph^+^ and Ph^−^ ALL	Preclinical (cell lines, primary cells) Preclinical (cell lines, primary cells) Preclinical (cell lines, primary cells, xenografts)	[262,263,264]
**Midostaurin** **(PKC412)**	FLT3-ITD/KIT Type I inhibitor	FLT3 mutated AML, SM	Market authorization (Rydapt^®^, Novartis)	[207,222]
**Momelotinib** **(CYT-387)**	JAK2/JAK1/JAK3/ALK2 Type I inhibitor	MPN MM	2 Phase 3 Preclinical (cell lines, primary cells)	[259,260]
**Nilotinib** **(AMN107)**	BCR-ABL Type II inhibitor	CML, Ph^+^ ALL	Market authorization (Tasigna^®^, Novartis)	[203]
**NS-018**	JAK2/Src Type I inhibitor	MPN MM	Phase 1-2 Preclinical (cell lines, mouse model)	[254,255]
**NVP-BSK805**	JAK2 (JAK2^V617F^) Type I inhibitor	JAK2^V617F^-MPN	Preclinical (cell lines, mouse model)	[256]
**NVP-BVB808**	JAK2 Type I inhibitor	MPN	Preclinical (cell lines)	[257]
**NVP-CHZ868**	JAK2/TYK2/KIT/PDGF/VEGFR Type II inhibitor	MPN B-ALL	Preclinical (cell lines, primary cells, mouse models) Preclinical (cell lines, xenografts, PDX)	[238,239]
**ON044580**	JAK2/BCR-ABL Allosteric inhibitor	JAK2^V617F^-MPN BCR-ABL^T315I^-CML	Preclinical (cell lines, primary cells)	[258]
**Pacritinib** **(SB1518)**	JAK2/FLT3/IRAK1 Type I inhibitor	MF Lymphomas, CLL, LPD	Phase 3 Phase 1	[233,234]
**Ponatinib** **(AP24534)**	BCR-ABL and BCR-ABL mutants FGFR/PDGFR/Src/RET/KIT/FLT1 Type II inhibitor	CML, Ph+ ALL	Market authorization (Iclusig^®^, Ariad)	[204]
**Ruxolitinib** **(INCB18424)**	JAK2/JAK1/JAK3 Type I Inhibitor	MPN CML HL MM AML, ALL	Market authorization (Jakavi^®^, Novartis) Phase 1 (CT) Phase 2 Phase 1 (CT) Phase1/2 (terminated)	[227,228,229,230,231,232]
**Quizartinib** **(AC220)**	FLT3 Type II inhibitor	FLT3-mutated AML	Market authorization (Japan) (Vanflyta^®^, Daiichi-Sankyo)	[217,218]
**Sorafenib** **(BAY43-9006)**	FLT3/PDGFR/ VEGFR/c-KIT/RAF Type II inhibitor	FLT3-mutated AML ALL, AML, MDS	Phase 1 Phase ½ (CT) Phase 1	[208,209,210]
**Sunitinib** **(SU11248)**	PDGFR/ VEGFR/c-KIT/FLT3 Type I Inhibitor	FLT3-mutated AML	Phase 1 Phase ½ (CT)	[214,215]
**Tandutinib** **(MLN518)**	FLT3/PDGFR/KIT Inhibits type III receptor kinases	AML/MDS AML SM	Phase ½ Phase ½ (CT) Preclinical (cell lines)	[216,224]
**TG101209**	JAK2/FLT3 Type I inhibitor	MPN MM T-ALL	Preclinical (cell lines, primary cells, mouse models) Preclinical (cell lines) Preclinical (cell lines, primary cells)	[235,236,237]
**WP1066 and WP1034** **(AG490 analogs)**	JAK2 Phosphorylation and/or degradation	JAK2^V617F^-mutated MPN AML	Preclinical (cell lines, primary cells) Preclinical (cell lines, primary cells)	[265,266,267]
**XL019**	Pan-JAK. Type I inhibitor	MPN	Phase 1 (terminated)	[268]
**Natural compounds**				
**Avicin D**	JAK/STAT3 Inhibits P-Y/JAK1/2 phosphorylation Unknown mechanism	MM CTCL	Preclinical (cell lines) Preclinical (cell lines, primary cells)	[269,270]
**Butein**	STAT3. Inhibits P-Y Activates tyrosine phosphatase SHP1 Unknown mechanism	MM	Preclinical (cell lines)	[271]
**Capsaicin**	JAK1/Src/ STAT3. Inhibits P-Y/ Inhbits kinase activity Unknown mechanism	MM	Preclinical (cell lines, xenografts)	[272]
**Celastrol**	JAK/Src/STAT3. Inhibits P-Y/Kinase activity Unknown mechanism	MM	Preclinical (cell lines)	[273]
**Curcubitacin I** **(JSI-124)**	STAT3. Inhibits P-Y/P-S of STAT3 Unknown mechanism	B-ALL, CLL, lymphomas	Preclinical (cell lines, primary cells)	[274,275]
**Curcumin** **diferuloylmethane**	JAK/STAT3/STAT5. Inhibits P-Y/kinase activity Unknown mechanism	CML T-cell leukemia T-cell lymphoma HL MM	Preclinical (cell lines) Preclinical (cell lines) Preclinical (cell lines, primary cells) Preclinical (cell lines) Preclinical (cell lines)	[279,280,281,282,283]
**Honokiol** **(HNK)**	STAT3. Inhibits P-Y Activate SHP1 Unknown mechanism	AML	Preclinical (cell lines, primary cells)	[276]
**Piperlongumine**	STAT3. Inhibits P-Y and other pathways Binds to cysteine 712 of STAT3 Unknown mechanism	MM	Preclinical (cell lines, xenografts)	[277]
**Sulforaphane**	STAT5. Inhibits transcriptional activity. Deacetylase inhibitor Unknown mechanism	CML	Preclinical (cell lines)	[289]
**Synthetic molecules**				
**CDDO** **(Imidazolide)**	STAT3/ STAT5. Inhibits P-Y Unknown mechanism	MM	Preclinical (cell lines)	[285]
**E804** **(Indirubin derivative)**	SFK/STAT5 Inhibits P-Y/ Inhibits kinase activity Unknown mechanism	CML AML	Preclinical (cell lines, primary cells) Preclinical (cell lines, xenografts)	[286,287]
**FLLL3 (Curcumin derivative)**	JAK/STAT3. Inhibits P-Y Unknown mechanism	MM	Preclinical (cell lines)	[284]
**JQ1** **(Thienodiazepine derivative)**	STAT5. Inhibits transcriptional activity.BET inhibitor. Unknown mechanism	T-ALL	Preclinical (cell lines, primary cells)	[290]
**17f**	STAT5. Inhibits P-Y and expression. Unknown Mechanism	AML, CML	Preclinical (cell lines)	[292]
**Naphtoquinone (NPQ) derivative**	STAT5. Inhibits P-Y/ Unknown mechanism	CML	Preclinical (cell lines)	[291]
**Drug repositionning**				
**Niclosamide** **(Antiparasitic)**	STAT3. Inhibits P-Y Unknown mechanism	MM	Preclinical (cell lines, primary cells)	[295]
**Nifuroxazide** **(Antidiarrheic)**	JAK/STAT3. Inhibits P-Y Unknown mechanism	MM	Preclinical (cell lines, primary cells)	[294]
**Pimozide** **(Antipsychotic)**	STAT3/STAT5. Inhibits P-Y Unknown mechanism	CML MPN FLT3-mutated AML MM	Preclinical (cell lines, primary cells) Preclinical (cell lines) (CT) Preclinical (cell lines, mouse model) Preclinical (cell lines)	[79,196,298,299]
**PPAR** **γ ligands** **(Antidiabetic)** **(Thiazolinediones)**	STAT5. Inhibits *STAT5A* and *STAT5B* gene expression STAT3. Inhibits transcriptional activity	CM, Ph^+^ ALL MM	Preclinical (cell lines, primary cells) Phase 2 (CT) Preclinical (cell lines, primary cells)	[60,301,302,303]
**Pyrimethamine** **(Antiparasitic)** **(Antifolate)**	STAT3. Inhibits P-Y Unknown mechanism	AML CLL/SLL MM	Preclinical (cell lines, primary cells, xenografts) Phase ½ Preclinical (cell lines)	[196,296]

AML (acute myeloid leukemia), B- and T-ALL (B- and T-acute lymphoblastic leukemia), CLL (chronic lymphocytic leukemia), CTCL (cutaneous T cell lymphoma), CML (chronic myeloid leukemia), CTCL (cutaneous T cell lymphoma), ET (essential thrombocythemia), HL (Hodgkin’s lymphoma), LPD (lymphoproliferative disorders), myelodysplastic syndromes (MDS), MF (myelofibrosis), MCL (mantle cell lymphoma), MLBCL (mediastinal large B cell lymphoma), MM (multiple myeloma), MPN (myeloproliferative neoplasm), Ph^+^ and Ph^−^ ALL (Philadelphia chromosome-positive and -negative acute lymphoblastic leukemia); PV (polycythemia vera), SLL (small lymphocytic lymphoma), SM (systemic mastocytosis), CT (combination therapy).

### 4.3. Direct Inhibitors of STAT3 and STAT5 in Hematopoietic Cancers (Table 3)

The mechanisms of direct STAT3/5 inhibition include disruption of tyrosine phosphorylation, dimerization, nuclear translocation and/or DNA binding. Targeting the SH2 domain was, therefore, the main focus for the design and identification of selective inhibitors.

#### 4.3.1. Inhibitors Targeting the SH2 Domain

Dimerization is an essential step in the canonical functions of STAT3 and STAT5. Blocking this process seems to be an optimal solution to directly inhibit aberrant STAT3/5 signaling in hematopoietic cancers. The SH2 domain is not only required for dimer formation but also in the recruitment of STAT3/5 to tyrosine-phosphorylated receptor complexes. Therefore, initial strategies aiming to identify phosphopeptide (P-Y-peptide) inhibitors able to block SH2/P-Y interactions were pursued by several teams. This approach was first attempted by Turkson et al. who demonstrated that the peptide PY*LKTK (where Y* represents P-Y^705^) inhibits STAT3 dimerization and tumor cell growth [192]. P-Y-peptides derived from STAT3 docking sites of gp130 and other cytokine or growth factors receptors were also used to identify the P-Y-peptide YLPQTV as a potent blocker of STAT3 dimerization and DNA binding [304,305]. Although highly specific, peptides usually have poor membrane permeability, low stability, and consequently low biological activities. This prompted investigators to develop peptidomimetics using the tripeptide PY*L, the minimum P-Y-peptide sequence required for STAT3 inhibition. One of these peptidomimetics, ISS610 was shown to be more potent in disrupting STAT3 dimerization and DNA binding but still had poor membrane permeability [193]. Structural and computational analysis of the interaction between ISS610 and the STAT3 SH2 domain led to the development of the peptidomimetic molecule, S3I-M2001, having increased membrane permeability but similar capacity in inhibiting STAT3 DNA binding [306]. Despite hard work in developing peptides or peptidomimetics with potent STAT3-inhibitory activity, poor permeability and metabolic stability have precluded their clinical testing. For the same reason, no peptide/peptidomimetic inhibitors of STAT5 have been developed. Based on the proof of concept that the STAT3-SH2/pY-peptide interaction was amenable to targeting, small nonpeptidic compounds that could specifically bind to the SH2 domains of STAT3 or STAT5 have become attractive candidates. Here, we will discuss the SH2 domain inhibitors that have been used in preclinical studies and clinical trials for the treatment of hematologic malignancies.

Most of the small molecules targeting the SH2 domain of STAT3 were identified by structure-based high-throughput virtual screening. The available X-ray crystallographic data of both the monomer and dimerized STAT3 bound to DNA were essential for these in silico studies [307]. Among these molecules, S3I-201, STA-21, C188, and STX-0119 were shown to block the phosphorylation, dimerization, DNA binding, and transcriptional activity of STAT3 as well as growth and survival of cancer cells [308,309,310,311]. In modeling studies, S3I-201 was shown to bind to the STAT3-SH2 domain through its salicylic acid moiety, which is known as a P-Y mimetic [308]. However, Genetic Optimization for Ligand Docking (GOLD) studies indicated that binding of S3I-201 was suboptimal, and structure–activity relationship (SAR) analyses based on this “hit” compound were conducted to derive analogs with improved STAT3 inhibitory activity. One of these analogs, S3I-1757, bound to the SH2 domain of STAT3 but still had a modest potency for decreasing STAT3 DNA binding [312]. However, S3I-1757 embedded in nanoparticles that had been conjugated with monoclonal antibodies against CD38 (denoted as CD38-S3I-NP) was demonstrated to have some increased efficacy in suppressing P-Y-STAT3 and tumor growth in xenograft models of MM. Data describing this compound are presented in this Cancers issue [313]. Among various S3I-201 derivatives, SF-1-066 was found to be the most potent. SF-1-066 was shown to directly bind STAT3 and to inhibit P-Y-STAT3 and growth of AML and MM cell lines with greater potency than S3I-201 [314,315]. A family of sixteen sulfonamide analogs of SF-1-066 was synthetized and BP-1-102 (17o) was proved to be the most active compound in this series. BP-1-102 exhibited improved inhibition of P-Y-STAT3 in MM cell lines [316]. In addition, BP-1-102 blocked P-Y-STAT3 in B-ALL cells overexpressing HMGA1 chromatin remodeling proteins and suppressed their growth [94]. Growth inhibition induced by this salicylic acid-based inhibitor was also reported for T-ALL cell lines [94]. N-alkylated derivatives of both SF-1-066 and BP-1-102, such as 16i and 21h molecules, were demonstrated to inhibit STAT3 function and to induce MM cell apoptosis, but these molecules also lost selectivity toward STAT3 [317]. BP-5-087, another analog of SF-1-066, was identified by SAR-based drug design and compound library screening. BP-5-087 was shown to be a more potent salicylic acid-based STAT3 inhibitor than SF-1-066 and to overcome IM resistance in CML stem cells [318].

A library of phosphopeptide mimicking salicylic acid-based molecules targeting the STAT3-SH2 domain was also employed to identify potent and STAT5-selective inhibitors. Among compounds that were selected in the screening via fluorescence polarization (FP) assays, BP-1-108 was found to be the more potent STAT5 inhibitor [319]. BP-1-108 significantly reduced P-Y-STAT5 and exhibited growth inhibitory properties in CML and AML cell lines. In silico-based studies using BP-1-108 as a scaffold identified compound 13a (or AC-4-130) as a selective and first nanomolar inhibitor of the STAT5B-SH2 domain [320]. AC-4-130 did not exhibit off-target kinase activity and suppressed P-Y-STAT5 at low micromolar concentrations in CML and AML cell lines, as well as in primary AML cells [81]. AC-4-130 inhibited the growth of AML cells in vitro and tumor formation in xenograft models of AML. Furthermore, AC-4-130 was also shown to synergistically increase the cytotoxic effect of the JAK1/2 inhibitor ruxolitinib and the p300/pCAF inhibitor garcinol in FLT3-ITD^+^ AML cells [81]. IST5-002, another STAT5 inhibitor, was identified using in silico structure-based screening and medicinal chemistry. IST5-002 was demonstrated to bind to the SH2 domain of STAT5B, to inhibit P-Y-STAT5 and to reduce the growth and viability of sensitive and IM-resistant CML and Ph^+^ALL cell lines [321]. Patient-derived CML or newly diagnosed and relapsed/TKI-resistant Ph^+^ALL cells were also sensitive to IST5-002 treatment. Moreover, IST5-002 was found to reduce leukemia development in PDX models of Ph^+^ALL [90]. A chromone-derived acyl hydrazine compound, identified through a high-throughput FP screen, was the first inhibitor to directly target the SH2 domain of STAT5B. However, high concentrations of this compound was required to disrupt STAT5 DNA binding and P-Y-STAT5 in CML and Burkitt’s lymphoma (BL) cell lines, respectively [322]. The same group next identified catechol-biphosphate as a selective inhibitor of the STAT5B SH2 domain. One of the catechol-biphosphate derivatives, stafib-1, was shown to be a nanomolar inhibitor of the STAT5B SH2 domain with more than 50-fold selectivity over the STAT5A-SH2 domain [323]. SAR studies based on the Stafib-1 compound and rational design led to the development of Stafib-2 with improved and high selective activity against STAT5B in CML cells [324].

Structure-based high-throughput virtual screening of four different chemical libraries followed by cell-based reporter assays identified STA-21 (or deoxytetrangomycin) as a new SH2 domain-targeting inhibitor of STAT3 [309]. STA-21 induced apoptosis of primary cells from chronic lymphoproliferative disorders of natural killer cells (CLPD-NK) and T-LGL patients and DLBCL cells when associated with YM155, a survivin suppressant [325,326]. A structural analog of STA-21, LLL-3, was developed to improve the cellular permeability of STA-21. LLL-3 was shown to promote apoptosis of CML cell lines and to synergistically act with IM to suppress CML cell growth and survival [327]. LLL-3 was further optimized by replacing its acetyl group with a sulfonamide to produce LLL-12, which prevented the phosphorylation of STAT3 and induced apoptosis in MM cell lines and primary MM cells in vitro, even in samples from patients with relapsed/refractory MM. LLL-12 also suppressed tumor formation in xenograft models of MM [328]. However, LLL-12 was required in high concentrations for in vivo studies due to low bioavailability. C188 compound was identified by virtual ligand screening of eight chemical libraries [310]. C188 was shown to inhibit P-Y-peptide binding to the SH2 domain of STAT3 by surface plasmon resonance (SPR) assays. Using C188 as the scaffold, C188-9 was identified in a hit-to-lead program and was shown to bind to the STAT3-SH2 domain with greater potency than the C188 compound. C188-9 effectively suppressed G-CSF-induced P-Y-STAT3 in AML cell lines and patient samples without affecting JAK or Src kinases. C188-9 also induced apoptosis and blocked the clonogenic growth of AML cells [329]. In silico screening studies followed by STAT3-dependent luciferase reporter gene assays led to the identification of STX-0119, another STAT3 inhibitor impacting the growth of lymphoma cell lines. Oral administration of STX-0119 was shown to reduce STAT3 target gene expression and to induce apoptosis in a xenograft model of lymphoma [311]. The STAT3-SH2 domain inhibitor STATTIC was identified by screening chemical libraries in an FP-based binding assay. This compound was demonstrated to selectively inhibit the dimerization and transcriptional activity of STAT3, although a report indicated that it also blocks STAT1 phosphorylation [330]. STATTIC reduced P-Y-STAT3 and viability of MM cells in three-dimensional (3D) culture and sensitized them to bortezomib, a proteasome inhibitor that is clinically used in MM treatment (this issue) [331]. Moreover, STATTIC suppressed growth and survival of NK/T cell lymphoma cell lines expressing the STAT3^Y640F^ mutant [332]. Among the STAT3-SH2 domain targeting inhibitors, OPB-31121 and OPB-51602 were the only compounds to have reached early phase clinical trials for treatment of hematopoietic malignancies [333]. OPB-31121 was shown to inhibit P-Y-STAT3 and P-Y-STAT5 without affecting upstream kinase activity in various myeloid leukemia cell lines expressing BCR-ABL, FLT3-ITD, or JAK2^V617F^, as well as in BL and MM cell lines [334]. Treatment with OPB-31121 also induced growth inhibition of these hematopoietic malignant cells and reduced tumor formation in PDX models of ALL, CML, and AML. Computational docking and molecular dynamics simulations (MDS) demonstrated that OPB-31121 bound with high affinity to the SH2 domain of STAT3 [335]. In the same way, OPB-51602 was also shown to interact with high affinity with the STAT3 SH2 domain to inhibit P-Y-STAT3 and to be effective against MM, BL, and AML cells in preclinical in vitro and in vivo studies [336]. Although promising data were obtained from these preclinical studies, clinical trials were terminated for both of these inhibitors due to poor pharmacokinetic properties, significant toxicity and lack of antitumor activity. Very recently, SD-36, a proteolysis targeting chimera (PROTAC) that selectively degrades STAT3 protein has been developed. This molecule consists of the cell-permeable STAT3-SH2 domain-targeting inhibitor, SI-109, conjugated to a ligand of Cereblon, an important component of the E3 ubiquitin ligase complex that is involved in ubiquitination and degradation of multiple cellular proteins. The resulting PROTAC binds to and recruits both STAT3 and the E3 ligase to form a productive ternary complex for ubiquitination and degradation. SD-36 was shown to induce STAT3 degradation, cell growth arrest, and apoptosis in lymphoma and leukemia cells lines as well as tumor regression in multiple xenograft mouse models of leukemias and lymphomas [337]. Lastly, several plant-derived molecules were also demonstrated to be direct inhibitors of STAT3. Using computational modeling and docking simulations, Withaferin A, a natural product isolated from the medicinal plant *Withania somnifera*, was found to interact with the SH2 domain of STAT3. Consequently, Withaferin A prevented IL-6–mediated or persistently activated P-Y-STAT3 and induced apoptosis in MM cells [338]. In a similar vein, YL064, a derivative of Sinomenine, a plant component that has been used to treat rheumatic diseases, was shown to target the SH2 domain of STAT3 and to induce MM cell death [339].

#### 4.3.2. Inhibitors Targeting the DNA Binding Domain (DBD)

STAT3/5 bind to specific DNA response elements within promoters to mediate transcriptional activation of target genes. Concerted efforts were therefore made to identify specific inhibitors targeting the DBD of STAT proteins. Most of the DBD inhibitors presented in this section were historically used against STAT3 and proved to be effective in some hematopoietic cancers.

Platinum (IV) compounds such as CPA-1, CPA-7, and platinum (IV) tetrachloride, were shown to inhibit STAT3 DNA binding activity [340]. IS3 295, a member of this class of molecules, was identified by screening of the NCI 2000 diversity set using electrophoretic mobility shift assays (EMSA). IS3 295 was demonstrated to irreversibly bind to the DBD of STAT3 in its active and inactive form and to prevent its interaction with specific response elements. This compound inhibited the constitutive DNA binding of STAT3 and induced apoptosis in MM cells [341]. Peptide aptamers as inhibitors of STAT3 represent one of the effective approaches to disrupt STAT3 DNA binding. The DBD-directed peptide aptamer DBD-1 was identified in yeast two-hybrid screenings, and its cell-penetrating form DBD-1-9R was shown to inhibit STAT3 DNA binding in EMSAs and to induce growth inhibition and apoptosis in MM cells [342].

Another class of STAT DBD inhibitors being used are duplex ODN (decoy oligonucleotides or dODN) and GQ-ODN. dODNs targeting STAT proteins are double-stranded DNA molecules mimicking the consensus STAT DNA binding sequence. These duplex ODNs act by competitively inhibiting the DNA binding of STAT proteins to their endogenous promoter elements thereby preventing their nuclear function [195]. For instance, a STAT5 dODN was shown to block DNA binding and transcriptional activity of STAT5 and to induce growth arrest and apoptosis in CML cell lines [343]. STAT3 dODN, and the second-generation cyclic STAT3 decoy (CS3D) with a longer half-life, were only tested in solid tumors and will not be discussed here. However, a dual-function molecule CpG-STAT3 dODN, which consists of a STAT3 dODN fused to the TLR9 agonist cytosine guanine dinucleotide (CpG), was reported to induce growth-inhibitory and immune-mediated effects against AML and DLBCL [74,344]. These compounds are rapidly internalized by TLR9^+^ immune and malignant cells to block the oncogenic activity of STAT3 and to promote an antitumor immune response. Both TLR9 stimulation and concurrent STAT3 inhibition were critical for immune-mediated therapeutic effects. GQ-ODN are G-rich oligodeoxynucleotides that form intra- and inter-molecular four-stranded structures [195]. For example, the GQ-ODN T40214 was shown to inhibit IL-6–induced P-Y-STAT3 and STAT3-mediated transcription [345]. Computer-simulated docking studies indicated that GQ-ODNs interact mainly with the SH2 domain of STAT3 and are able to disrupt STAT3 dimers bound to DNA. Blocking STAT3 with the GQ-ODN T40214 loaded into nanoparticles was shown to be effective in a mouse model of T-ALL [346]. However, the use of GQ-ODN remains problematic due to the large size and potassium-dependence of this molecular probe.

**Table 3:** Direct inhibitors of STAT3 and STAT5 that have been tested in hematologic cancers.

Target	Drugs	Hematologic Malignancy	Stage of Clinical Development	References
**Direct STAT3 inhibitors**				
**mRNA**	AZD9150 (IONIS-STAT3Rx)	AML, MDS DLBCL, HL, NHL	Preclinical (cell lines, primary cells PDX) Phase 1	[347,348,349]
	CpG-STAT3-siRNA	AML, MM	Preclinical (cell lines, primary cells, mouse model of AML, xenografts)	[73,350]
**SH2 domain**	BP-5-087, BP-1-102/17o (S3I-201 derivatives)	CML B- ALL, T-ALL MM	Preclinical (cell lines, primary cells) Preclinical (cell lines, xenografts) Preclinical (cell lines)	[94,316,317,318]
	CD38-S3I-NP (S3I-1757)	MM	Preclinical (cell lines, xenografts)	[313]
	C188-9	AML	Preclinical (cell lines, primary cells)	[329]
	LLL-3	CML	Preclinical (cell lines)	[327]
	LLL-12	MM	Preclinical (cell lines, primary cells, xenografts)	[328]
	OPB-51602	MM, NHL, AML, CML	Phase 1 (terminated)	[336]
	SF-1-066 (S3I-201 derivative)	AML, MM	Preclinical (cell lines)	[314,315]
	STA-21	CLPD-NKs T-LGL DLBCL	Preclinical (primary cells) Preclinical (primary cells) Preclinical (cell lines)	[325,326]
	STATTIC	MM NK lymphomas T-cell lymphomas	Preclinical (cell lines) Preclinical (primary cells) Preclinical (primary cells)	[331,332]
	STX-0119	NHL	Preclinical (cell lines, xenografts)	[311]
	SD-36	AML, ALCL	Preclinical (cell lines, xenografts)	[337]
	YL064	MM	Preclinical (cell lines, primary cells)	[339]
	Withaferin A	MM	Preclinical (cell lines)	[338]
**DBD**	CpG-STAT3dODN (decoy oligonucleotides)	AML DLBCL	Preclinical (cell lines, xenografts, mouse models) Preclinical (cell lines, xenografts, mouse models)	[74,344]
	DBD-1-9R (peptide aptamer)	MM	Preclinical (cell lines)	[342]
	IS3-295 (Platinum IV)	MM	Preclinical (cell lines)	[341]
	T40214 (GQ-ODN)	T-ALL	Preclinical (mouse model)	[346]
**Direct STAT5 inhibitors**				
**SH2 domain**	AC-4-130	AML, CML	Preclinical (cell lines, primary cells, xenografts)	[81,320]
	BP-1-107, BP-1-108	AML, CML	Preclinical (cell lines)	[319]
	IST5-002	CML Ph+ ALL	Preclinical (cell lines, primary cells) Preclinical (cell lines, primary cells, PDX)	[90,321]
	Stafib-1	CML	Preclinical (cell lines)	[323]
	Stafib-2	CML	Preclinical (cell lines)	[324]
**DBD**	STAT5 dODN	CML	Preclinical (cell lines)	[343]
**Dual STAT3/STAT5 inhibitors**				
**SH2 domain**	OPB-31121	CML, AML, ALL, BL, MM, NHL, MM	Preclinical (cell lines, xenografts, PDX) Phase 1 (terminated)	[334]

AML (acute myeloid leukemia), ALCL (anaplastic large cell lymphoma), B-ALL/T-ALL (B- or T- acute lymphoblastic leukemia), Ph^+^ ALL (Philadelphia chromosome-positive acute lymphoblastic leukemia), BL (Burkitt’s lymphoma), CLPD-NKs (chronic lymphoproliferative disorders–natural killer cells), CML (chronic myeloid leukemia), DLBCL (diffuse large B cell lymphoma), HL (Hodgkin’s lymphoma), MDS (myelodysplastic syndrome), MM (multiple myeloma), NHL (non-Hodgkin’s lymphoma), T-LGL (T cell large granular lymphocytic) leukemia.

#### 4.3.3. Inhibitors Targeting STAT3/5 mRNAs

Oligonucleotide-based inhibition of STAT3 and STAT5 has also been achieved by using antisense oligonucleotides (ASO) or mRNA knockdown (siRNA) [195]. Although siRNAs and ASOs were widely used to illuminate the oncogenic activity of STAT3 and STAT5 in hematopoietic malignant cells, few of these molecules were developed as anticancer therapeutic agents. We will discuss here two examples of ASOs and siRNAs that gave promising results in preclinical studies and clinical trials. The first example is the chemically modified ASO, AZD9150, which specifically targets STAT3 mRNA and is now in phase 1/2 clinical trials. AZD9150, without any delivery agent, was shown to inhibit STAT3 expression in primary AML/MDS leukemia stem cells and to inhibit leukemic cell growth in vitro and in vivo using PDX models of AML/MDS [347]. Inhibitory effects of AZD9150 on STAT3 expression and cell growth were also demonstrated in preclinical models of lymphomas [348]. Furthermore, AZD9150 was well tolerated and demonstrated efficacy in patients with highly treatment-refractory lymphoma [349]. The second example is a STAT3 siRNA conjugated to the TLR9 ligand CpG described above. STAT3 silencing mediated by this CpG-siRNA inhibited tumor growth in xenograft models of AML or MM [350]. In an elegant study, Hossain et al. demonstrated that the CpG-STAT3 siRNA conjugate stimulates systemic antitumor immunity and antigen-specific activation of CD8^+^ T cells in a mouse model of AML [73]. Intravenous administration of CpG-STAT3 siRNA showed a direct immunogenic effect on leukemic cells indicating that targeted STAT3 inhibition and TLR9 triggering blocks leukemia cell growth by promoting antitumor immunity rather than direct tumor cell killing. To date, no inhibitors targeting STAT5A/STAT5B mRNAs were developed for therapeutic purposes.

## 5. STAT3/5 Inhibitors in Hematopoietic Cancers: Inherent Limitations and Future Challenges

Despite intensive efforts made during the last 15 years, clinically applicable STAT3/5 inhibitors still remain elusive. Toxicity, poor tumor targeting, lack of cell penetrance and rapid degradation are some examples of the problems to be considered and addressed. There are also several other reasons that could hinder the development of such inhibitors. First, STAT3 and STAT5 display multifaceted activity due to their canonical and non-canonical functions in cancer cells. The definition of constitutively active STAT3/5 only based on P-Y-STAT3 and/or P-Y-STAT5 levels as described in chapter 3 may not be broadly representative. For example, the use of decoy ODNs or SH2 domain-targeting inhibitors would not be effective in CLL cells, in which a predominant mitochondrial localization and function of P-S^727^-STAT3 has been evidenced [102]. Furthermore, it has been shown that nuclear translocation and DNA binding of STAT3 or STAT5 can occur independently of their P-Y status. These observations indicate that SH2 domain-targeting inhibitors may not be sufficient to fully abrogate STAT3/5 oncogenic functions, which may contribute to the limited success of these compounds. Second, extensive crosstalk and alternative signaling pathways present in TKO-driven hematopoietic malignancies probably render single-agent STAT3/5 inhibition less effective. Third, adverse effects of STAT3/5 inhibitors may be associated with the physiological function of these proteins in normal tissues including hematopoietic and lymphoid tissues, resulting in STAT3- or STAT5-specific toxicity. Previous studies indicated that STAT5-deficient NK cells induce angiogenesis and promote tumor progression, highlighting the potentially detrimental effects of STAT5 inhibitors on NK cell-mediated tumor immune surveillance [37]. Moreover, STAT3 and STAT5 were also shown to activate tumor suppressor pathways, strengthening the possible adverse effects of STAT3/5 inhibitors. Most of these tumor suppressor functions were described in different reviews including one published as part of this special issue [8,32]. One of the tumor suppressor activities of STAT3 is linked to the level of STAT3β expression, which could explain why different AML patient subsets, based on the STAT3β/STAT3α mRNA ratio, are more or less sensitive to STAT3 inhibitors [34]. In such a case, STAT3 direct inhibitors would also target STAT3β which is unwanted for AML treatment. Despite these limitations, optimization of currently available STAT3/5 inhibitors and discovery of new compounds that specifically target other functional domains such as the NH2-terminal domain, domains involved in the subcellular localization of STAT3/5 or STAT3/5/Rac1 interacting domains, as well as drugs activating negative regulators of STAT3/5 such as SOCS and PIAS proteins, still have to be considered in the future. Although most of the direct inhibitors have shown a moderate efficacy in treating hematologic cancers as single agents, they act synergistically with clinically used chemotherapeutic drugs or TKIs. Importantly, targeting STAT3 and/or STAT5 signaling was demonstrated to overcome drug resistance in hematopoietic malignant cells, suggesting that combination therapy using STAT3/5 inhibitors is the most attractive approach to fight against relapsed hematopoietic malignancies. For example, STAT3 and STAT5 were both recognized as important effectors of de novo and acquired IM-resistance in CML cells [57,60,62]. In fact, activation of STAT3 was demonstrated to be an important positive autocrine-paracrine feedback loop in the therapeutic treatment of oncogene-addicted cancer cells [351]. Activation of STAT3/5 elicited by bone marrow microenvironment-derived signals was found to mediate resistance of CML cells to IM treatment, and inhibition of STAT3/5 suppressed IM-resistant CML cells in the niche. In both cases, extrinsic activation of STAT3 or STAT5 was induced by JAK kinases, and the JAK1/2 inhibitor ruxolitinib was shown to act synergistically with IM in killing resistant CML cells [62,229]. In a similar vein, the STAT5-SH2 domain inhibitor AC-4-130 increased the cytotoxicity of ruxolitinib in AML cells [81]. Combined targeting of STAT3 and STAT5 is another approach to overcome TKI resistance in CML cells. For example, the triterpenoid CDDO-Me (bardoxolone methyl) was shown to act synergistically with IM to kill resistant BCR-ABL-expressing cells [63]. In addition, blocking STAT3 and/or STAT5 with natural compounds or drugs that have been proven to be safe in humans might help to reduce the side effects of combination therapies. For example, piperlongumine (Table 2) enhanced the anti-MM effect of the proteasome inhibitor bortezomib, while pimozide increased the cytotoxic effects of TKIs in myeloid leukemias [277,298]. Besides its oncogenic activity, STAT3 is a central immune checkpoint regulator in cancer cells and tumor-associated immune cells. Oncogenic STAT3 drives PD-L1 expression in lymphoma and AML cells and may promote tumor immune evasion by inducing an immunosuppressive microenvironment [73,114]. In this context, the combination of STAT3 inhibitors with anti-checkpoint antibodies blocking PD-L1/programmed cell death 1 (PD-1) interactions might be a promising therapeutic approach for lymphomas and leukemias. Collectively, these data indicate that combination therapies using inhibitors that indirectly or directly target STAT3 and/or STAT5 might provide new therapeutic opportunities for relapsed or refractory hematopoietic malignancies.

## 6. Conclusions

The design and development of STAT3/5 inhibitors evolved rapidly during the last 10 years. Computer simulations and other in silico studies such as high-throughput virtual screening, cell-based assays, biophysical and biochemical approaches led to the identification of selective STAT3/5 inhibitors with potent anticancer effects. STAT3/5 inhibitors are likely to become a valuable addition to the expanding arsenal of drugs against hematopoietic cancers and solid tumors. However, there are some limitations to this relative success story that must be taken into account. Optimization of STAT3/STAT5 inhibitors by chemical modifications and drug delivery systems are both required for resolving issues linked to stability, cell permeability, and targeted delivery to tumor cells. The development of antibodies conjugated to STAT3/5 inhibitors or antibody-conjugated nanoparticles harboring STAT3/5 inhibitors might be promising but remains challenging. Identification of new inhibitors targeting the non-canonical functions of STAT3/5 is also desirable in treating some hematologic malignancies. Last but not least, the optimization of combination therapies using STAT3/5 inhibitors with molecules targeting tyrosine kinases or other key players in cancer will be required for finding the right combination that safely unlocks drug resistance in hematologic cancers.

## Figures and Tables

**Table 1:** STAT3 and STAT5 in hematologic cancers.

Hematopoietic Lineage	Hematologic Malignancies		Contribution of STAT3 and/or STAT5A/5B		
Myeloid compartment	Ph^+^ MPN	**CML (BCR-ABL)**	STAT3	STAT5A	STAT5B
	Ph^−^ MPN	PV JAK2^V617F^	STAT3	STAT5	
		ET JAK2^V617F^			
		PMF JAK2^V617F^			
		SM KIT^D816V^			
	AML	Flt3-ITD		STAT5	
		CBF-AML Kit^D816V^	STAT3	STAT5	
		APL	STAT3	STAT5	
		t (15;17)			
Lymphoid compartment	ALL	Pre-B-ALL	STAT3	STAT5	
		B-ALL			
		T-ALL	STAT3	STAT5A	STAT5B
	T-LGL Leukemias		STAT3	STAT5A	STAT5B
	CLL		STAT3		
	Lymphomas	HL	STAT3	STAT5	
		DLBCL	STAT3	STAT5	
		ALCL	STAT3		STAT5B
		γδ-T cell Lymphomas	STAT3		STAT5B
		NK-T cell Lymphomas	STAT3		STAT5B
	Multiple Myelomas		STAT3		

Changes in font size and bold text refer to the level of contribution of each STAT protein in the disease. ALCL (anaplastic large cell lymphoma), AML (acute myeloid leukemia), APL (acute promyelocytic leukemia), B- or T-ALL (B- or T- acute lymphoblastic leukemia), CBF-AML (core binding factor-acute myeloid leukemia), CLL (chronic lymphocytic leukemia), CML (chronic myeloid leukemia), DLBCL (diffuse large B cell lymphoma), ET (essential thrombocythemia), Flt3-ITD (FMS-like tyrosine kinase 3-internal tandem duplication), HL (Hodgkin lymphoma), Ph^+^MPN and Ph^−^MPN (Philadelphia chromosome-positive and Philadelphia chromosome-negative myeloproliferative neoplasm), NK (natural killer cell), PMF (primary myelofibrosis), PV (polycythemia vera), SM (systemic mastocytosis), and T-LGL (T cell large granular lymphocytic) leukemia.
